# H3 Subtype Avian Influenza Virus: Ecology, Cross-Species Evolution, and Public Health Threats

**DOI:** 10.3390/microorganisms14102240

**Published:** 2026-10-03

**Authors:** Kaihui Cheng, Zhijun Yu

**Affiliations:** 1 Institute of Animal Science and Veterinary Medicine, Shandong Academy of Agricultural Sciences, No. 23788 of Gongye North Road, Jinan 250100, China; chengkaihui@126.com; 2 Poultry Institute, Shandong Academy of Agricultural Sciences, No. 23788 of Gongye North Road, Jinan 250100, China; 3 Shandong Provincial Key Laboratory of Livestock and Poultry Breeding (PKL2024B15), No. 23788 of Gongye North Road, Jinan 250100, China

**Keywords:** H3 subtype avian influenza virus, genetic evolution, cross-species transmission, mammalian adaptation, zoonotic spillover, reassortment, public health risk

## Abstract

H3 subtype avian influenza virus (AIV) is one of the most widely distributed and frequently detected low-pathogenicity avian influenza viruses (LPAIVs) in wild birds and poultry worldwide. Wild waterfowl are the natural reservoir of this virus. In recent years, driven by frequent genetic reassortment and continuous evolution, H3 subtype AIV has expanded its host range to include various mammalian species—dogs, cats, horses, seals, pigs (experimental infection), and humans. Among these, the H3N8 subtype shows the broadest cross-species transmissibility, having established sustained transmission lineages in horses and dogs, while also causing sporadic infections in seals and humans. Avian-origin H3N2 canine influenza virus has circulated stably in dog populations across Asia and North America for nearly two decades. Between 2022 and 2023, China reported three human cases of H3N8 subtype AIV infection, one of which was fatal (Guangdong, March 2023), indicating that this virus poses a potential zoonotic transmission risk. Novel triple-reassortant strains, including genotype G25 of H3N8 and H3N3, first detected in chicken flocks in late 2022, have continued to emerge. Their HA genes originate from H3 subtype viruses of wild bird or chicken origin, whereas their internal gene segments are predominantly acquired from H9N2 subtype AIV, resulting in enhanced mammalian adaptability. This review summarizes the global epidemiology of H3 subtype AIV (covering wild birds, poultry, and live poultry markets), its genetic evolution and reassortment patterns, and the molecular mechanisms underlying cross-species transmission. This review particularly highlights the regulatory roles of key amino acid mutations—such as PB2-D701N, M1-M192V, and HA-Q226L/G228S—in receptor binding preference, polymerase activity, and pathogenicity. This review also summarizes recent advances in detection techniques (multiplex RT-PCR, quantum dot-based immunochromatographic strips, RT-RAA, and CRISPR-Cas13a) and vaccine development (inactivated, adenovirus-vectored, and mRNA vaccines). Key prevention and control challenges—including covert circulation, surveillance gaps, and limited subtyping diagnostic capacity at primary laboratories—are also discussed. This review proposes that H3 subtype AIV should be recognized as a potential zoonotic threat warranting continued surveillance, although no evidence of sustained human-to-human transmission has been documented to date. Better understanding of cross-species mechanisms, stronger surveillance and early warning, and evidence-based control strategies are needed to inform future risk assessment.

## 1. Introduction

Avian influenza virus (AIV), a member of the genus *Alphainfluenzavirus* within the family Orthomyxoviridae, possesses a segmented genome comprising eight single-stranded negative-sense RNA segments. Based on the antigenic diversity of the two surface glycoproteins, hemagglutinin (HA) and neuraminidase (NA), influenza A viruses are currently classified into 18 HA subtypes (H1–-H18) and 11 NA subtypes (N1–N11). Among these, subtypes H1–H16 and N1–N9 are maintained in wild waterfowl, their natural reservoir hosts, circulating continuously among wild birds and poultry. In contrast, subtypes H17N10 and H18N11 have been exclusively identified in bats, exhibiting distinct host tropisms [1].

Among all AIV subtypes, the H3 subtype demonstrates an exceptionally broad host tropism, infecting wild waterfowl, poultry, and multiple mammalian species, including dogs, cats, seals, horses, pigs (under experimental conditions), and humans [2]. H3 subtype AIV is classified as a low-pathogenicity avian influenza virus (LPAIV), typically not causing acute mass mortality in poultry; however, its long-term colonization and continuous circulation in poultry populations impose persistent economic losses on the poultry industry.

Owing to its broad host adaptability and the evolutionary advantages conferred by frequent genetic mutation and reassortment, the H3 subtype AIV has attracted increasing attention from global public health systems [3]. The 1968 Hong Kong H3N2 influenza pandemic marked a landmark public health event in which H3 subtype AIV breached the species barrier, triggering a large-scale human pandemic [4]. Subsequently, H3N2 subtype influenza viruses established stable transmission in humans and have now become one of the seasonal influenza viruses [5]. In 2022, the first human case of H3N8 subtype AIV infection was reported globally in China [6,7]; concurrently, novel reassortant H3N8 subtype AIV variants were detected in chicken flocks in Hong Kong [8]. These converging epidemiological findings provide compelling evidence that the H3 subtype AIV possesses prominent cross-species transmissibility and zoonotic potential, posing a realistic threat to public health security.

The primary objectives of this review are: (1) to systematically summarize the global epidemiological patterns of H3 subtype AIV across wild birds, poultry, and mammalian hosts; (2) to analyze the genetic evolution and reassortment events that have generated novel variant strains, with emphasis on H3N8 and H3N3 triple-reassortant viruses and other H3NX subtypes; (3) to elucidate the molecular mechanisms underlying cross-species transmission, including the coordinated effects of adaptive mutations across different gene segments on receptor-binding preference and pathogenicity; and (4) to examine current detection methods, surveillance systems, and vaccine platforms, and to propose integrated control strategies and future research directions.

Relevant literature for this review was identified by searching the PubMed database for articles published between 2016 and 2026, using search terms including “H3 subtype avian influenza,” “H3N8,” “H3N3,” “cross-species transmission,” “mammalian adaptation,” “receptor binding,” “surveillance,” and “vaccine”. From the retrieved results, studies directly relevant to the objectives of this review were selected, with priority given to recent advances published within the last three years. Additional relevant studies were also identified by manually screening the reference lists of retrieved articles.

To avoid conceptual ambiguity, the following key terms are defined as used throughout this review. Mutation refers to point changes in nucleotide sequences; reassortment denotes the exchange of entire gene segments between co-infecting viruses. Positive selection indicates the preferential fixation of beneficial amino acid changes driven by host adaptation, whereas genetic drift describes the gradual accumulation of random mutations over time. Antigenic drift is used specifically for mutations in surface glycoproteins (particularly HA) that alter antibody recognition, and convergent evolution describes the independent acquisition of similar adaptive mutations in different viral lineages under comparable host selection pressures.

This review summarizes recent research advances on the H3 subtype AIV, covering global epidemiological characteristics, genetic evolution patterns, cross-species transmission and mammalian adaptation, pathogenicity, virus surveillance, vaccine development, and comprehensive prevention and control strategies. This review aims to provide a theoretical reference for future research and scientifically based prevention and control of this virus.

## 2. Global Epidemiology of H3 Subtype AIV

### 2.1. Geographic Distribution and Host Range

H3 subtype AIV is globally distributed. Based on analyses of global surveillance databases, the host range of this subtype covers at least 90 species of wild birds and poultry; Alaska, Central Asia, and multiple provinces in China represent key hub regions for large-scale transmission and regional epizootics of H3 subtype AIV [9].

Wild birds serve as the natural reservoir hosts for H3 subtype AIV [10,11] (Table 1). In addition to wild birds and poultry, H3 subtype AIV can cross species barriers to infect various terrestrial mammals. Among these, the H3N8 subtype is the most frequently detected subtype in horses, while the H3N2 subtype circulates extensively in dogs [11], and H3N2 subtype strains also predominate in cats [12]. Of particular concern, among H3 subtype AIVs, the H3N8 subtype exhibits the strongest cross-species transmissibility and the broadest host range, having been isolated from wild birds, poultry, horses, dogs, seals, pigs (experimental infection), and humans [7,11,13,14] (Table 1).

Beyond Asia, long-term surveillance in North America has documented H3-subtype AIV circulation among wintering waterfowl along the Texas Gulf Coast; H3N8 ranked among the most commonly recovered subtype combinations in the 2006–2007 sampling season [30]. In Europe, surveillance targeting Passeriformes in Ukraine detected H3N8 in great tits (*Parus major*). Phylogenetic reconstruction placed this virus within the Eurasian H3N8 lineage, showing close genetic relatedness to viruses of the same subtype circulating in wild waterfowl [31]. In Central Asia, active wild-bird surveillance in Kazakhstan identified H3N8 among prevalent AIV subtypes, with genomic segments belonging to Asian, European, and Australian lineages co-circulating. These findings underscore Central Asia’s role as a migratory transmission hub connecting East Asian and European flyways [32,33]. Similarly, surveillance conducted in Mongolia between 2021 and 2023 recovered H3N8 as the predominant subtype, accounting for 39.0% of all isolated AIVs, and these isolates shared close genetic relationships with viruses circulating in wild birds in Korea [34]. In Australia, nationwide wild-bird surveillance (2005–2007) recovered H3N8, albeit with lower overall PCR-positive prevalence (1.0%) relative to reports from Northern-Hemisphere sites [35]. Collectively, these datasets illustrate the broad geographic distribution of H3-subtype AIV, yet surveillance intensity, sampling continuity, and laboratory methodologies differ markedly across regions—a limitation that highlights the need for harmonized global monitoring frameworks.

**Figure 1:**
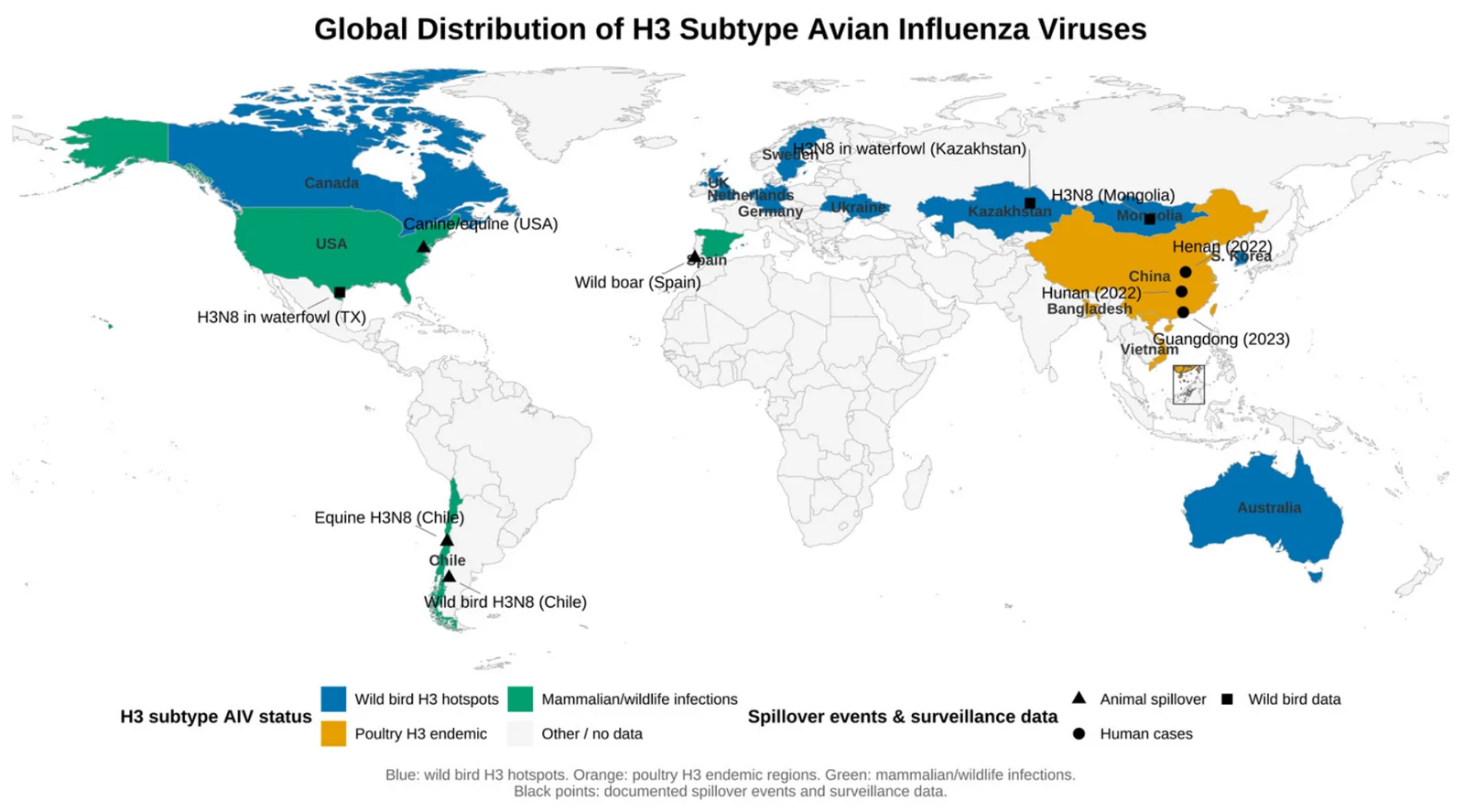
Global distribution of H3 subtype avian influenza viruses across different host species. Blue indicates regions with documented H3 subtype circulation in wild birds, including Canada, Mongolia, Kazakhstan, South Korea, the United Kingdom, Germany, the Netherlands, Sweden, Ukraine, and Australia. Orange indicates endemic regions in poultry, primarily China, Vietnam, and Bangladesh. Green indicates regions where mammalian or wildlife infections have been documented, including the United States, Chile, and Spain. Black points indicate documented spillover events and surveillance data: circular points (●) represent human H3N8 cases (Henan 2022, Hunan 2022, Guangdong 2023); triangular points (▲) represent animal spillover events, including equine H3N8 and wild bird H3N8 in Chile, canine/equine H3N8 in the United States, and serological evidence of H3 subtype AIV exposure in wild boars in Spain; square points (■) represent wild bird surveillance data, including H3N8 detection in waterfowl in Texas (USA), H3N8 in waterfowl in Kazakhstan, and H3N8 in Mongolia.

Nevertheless, direct cross-study comparison of apparent detection rates is confounded by heterogeneous sampling strategies (e.g., targeted versus opportunistic surveillance, hunter-harvested versus live-captured birds), variable laboratory workflows (virus isolation versus real-time RT-PCR), and divergent seasonal sampling windows, all of which exert substantial influence on observed prevalence estimates. Accordingly, these prevalence metrics should be interpreted qualitatively as evidence for widespread H3-subtype circulation across diverse avian ecological compartments, rather than treated as quantitative benchmarks for inter-regional comparison. The global distribution of H3 subtype AIV across different host species is summarized in Figure 1.

### 2.2. Prevalence in Wild Birds

Long-term surveillance of migratory blue-winged and green-winged teal in North America has demonstrated seasonal variation in the subtypes carried by waterfowl. H3 subtype AIV predominates during the autumn migration period, whereas other AIV subtypes become more prevalent in spring, exhibiting a clear seasonal fluctuation pattern [36]. Continuous surveillance of mallards in northwestern Minnesota from 2007 to 2016 revealed that H3 and H4 subtype AIVs consistently dominated local prevalence, collectively accounting for 65.7% of all HA subtype detections [37].

Long-term surveillance of wild birds in multiple European countries has consistently detected a high proportion of H3 subtype AIV strains. An eight-year site-specific surveillance program (2002–2009) of mallards in southern Sweden demonstrated that the H3 subtype was the predominant AIV subtype in waterfowl [10]. Surveillance data from 25 European countries indicated that H3 subtype AIV is commonly detected in wild birds, with H3N2 and H3N8 subtype AIVs circulating extensively among migratory waterfowl in Europe, with the United Kingdom, Germany, the Netherlands, and Italy being the primary countries of detection [11].

Surveillance in various Asian countries has likewise confirmed the widespread prevalence of H3 subtype AIV in wild birds. Surveillance of wild birds in Kazakhstan from 2002 to 2009 revealed that H3 and H13 subtype AIVs were the regional predominant subtypes [38]. A nationwide survey of wild birds in South Korea from 2012 to 2014 showed that H3 subtype AIV maintained a relatively high detection proportion [39]. Surveillance of wild birds in Mongolia from 2021 to 2023 demonstrated that H3N8 subtype AIV (39.0%) was the most frequently detected subtype [34].

In summary, H3 subtype AIV maintains a high prevalence level in wild waterfowl across all continents, representing one of the most active subtypes within the global wild bird influenza virus community.

### 2.3. Prevalence in Poultry

In recent years, the host distribution pattern of H3 subtype AIV within the poultry production ecosystem has undergone significant evolution. This has been characterized by a gradual shift in host preference from domestic waterfowl (ducks and geese) to terrestrial poultry (chickens), disrupting the previously relatively fixed transmission pattern. Historically, domestic waterfowl served as the natural reservoir hosts of H3 subtype AIV, whereas chickens experienced only sporadic infections that failed to establish sustained transmission chains [2]. However, over the past decade, continuous surveillance and whole-genome sequencing have confirmed that wild bird-origin H3 subtype AIVs have progressively enhanced their adaptation to terrestrial poultry following spillover into poultry populations. This adaptation occurs through the accumulation of genetic mutations and inter-subtype reassortment, ultimately achieving a host niche shift.

Surveillance data from China between 2009 and 2022 yielded 188 H3 subtype AIV isolates from domestic duck populations, which diverged into four stably established sublineages, encompassing 126 genotypes. This indicates that H3 subtype AIV has generated substantial genetic diversity within waterfowl hosts, providing a diverse mutational background for subsequent cross-host evolution and adaptive mutations [26]. Guangdong, Guangxi, and other southern provinces represent the core dissemination hubs for poultry-origin H3 subtype AIV in China, where domestic ducks dominated the early viral establishment and regional spread, laying an important foundation for subsequent adaptation to chicken hosts and nationwide dissemination. As the virus continued to evolve, detection rates of H3N3, H3N8, and other AIV subtypes in Chinese chicken flocks have increased continuously since 2021 (Table 1), reflecting a gradual host shift of H3 subtype AIV from domestic ducks to chickens [40]. Whole-genome tracing analyses further revealed that amino acid substitutions in the HA protein antigenic sites, together with reassortment and adaptation of internal genes with the genotype G57 of H9N2 subtype AIV, constitute the core molecular basis driving antigenic variation and significantly enhanced host adaptability of H3 subtype AIV in chickens [40].

Epidemiological surveillance data from poultry in Southeast Asia likewise confirm the continuous evolution and shifting host adaptability of H3 subtype AIV within poultry ecosystems. Long-term surveillance of poultry pathogens in Vietnam since 2011 has yielded 110 poultry-origin H3 subtype AIV isolates; phylogenetic analysis based on HA genes classified all isolates into eight independent genetic lineages, reflecting the highly complex genetic divergence of poultry-origin H3 subtype AIV in the region [24]. The year 2013 marked a critical turning point in local viral evolution: the original lineages II, III, and VI gradually declined, lineage I maintained its predominance, and lineages IV, V, and VIII emerged as new predominant circulating groups. Notably, strains from several circulating lineages (groups I–IV) showed close genetic relatedness to viruses detected in wild waterfowl, suggesting that wild waterbirds may serve as a continuing source of H3 subtype AIV in poultry in this region [40].

A cross-sectional epidemiological survey of poultry in Bangladesh further supports the host-shift trend of H3 subtype AIV. Data showed that the overall H3 subtype AIV positivity rate in the local poultry population was 3.4%, and although waterfowl still had a 5.71-fold higher infection risk than chickens, chicken flocks had established a detectable infection base [41]. This result indicates that waterfowl remain the natural dominant hosts for H3 subtype AIV, but host adaptability of the virus in chickens has significantly increased, with a clear trend of cross-host adaptation toward terrestrial poultry.

Synthesizing epidemiological investigations and genomic evidence from national and international sources, H3 subtype AIV has significantly breached traditional host barriers, establishing stable and extensive cross-host transmission cycles within poultry populations. The host distribution pattern has completed a transition from “waterfowl-single-host dominance” to “waterfowl–chicken dual-host co-circulation”. The evolutionary rate of H3 subtype AIV has increased markedly after invading the poultry ecosystem. The HA gene exhibits an evolutionary rate of 7.31 × 10^−3^ substitutions/site/year in chicken hosts, significantly higher than the 5.52 × 10^−3^ substitutions/site/year in duck hosts. These data indicate that chicken hosts provide stronger molecular driving forces for the rapid mutation and evolution of H3 viruses [40]. These estimates should nevertheless be interpreted with caution, because they stem from discrete phylodynamic models, with outputs conditional on molecular clock assumptions and sequence-sampling density. Given inconsistent reporting of confidence intervals among the original studies, direct cross-study comparisons should be avoided. Poultry-origin H3 subtype AIVs represented by the novel reassortant genotype G25 of H3N8 have acquired zoonotic potential, not only causing persistent economic losses to the poultry industry but also posing a prominent public health safety concern.

### 2.4. Live Poultry Markets (LPMs)

LPMs serve as critical hubs for AIV aggregation, reassortment, and cross-regional dissemination, playing a pivotal role in the transmission of H3 subtype AIV. Surveillance data from southern China between 2014 and 2021 demonstrated that H3 subtype AIV positivity rates in LPMs were generally elevated, with viral prevalence exhibiting significant regional differences and seasonal fluctuation patterns [42]. Peaks in AIV nucleic acid positivity occurred primarily during January and February each year, during which the detection proportions of H3, H4, H6, and other subtypes increased markedly [43]. Multiple ecological factors, including migratory bird migration patterns, inter-regional poultry trade and large-scale production cycles, and seasonal variations in environmental temperature and humidity, collectively shape the seasonal prevalence pattern of H3 subtype AIV.

Nationwide long-term surveillance data have revealed elevated prevalence proportions of H3 subtype AIV in certain sampling contexts. Long-term surveillance of environmental samples from LPMs in Guangxi, China, between 2016 and 2019 consistently detected H3, H4, H6, and H9 subtype AIVs [44]. Nationwide environmental surveillance in LPMs from 2019 to 2023 showed that the detection rate of the highly pathogenic H5 subtype had declined significantly. Meanwhile, the prevalence of LPAIVs represented by the H3 subtype increased continuously, while the H9 subtype maintained a persistently high prevalence level. These trends indicate that the AIV epidemic spectrum has undergone a clear shift [43]. From September 2021 to May 2022, large-scale sampling across 25 provinces in China collected a total of 38,639 samples. From these, 98 H3N8 subtype AIV strains were isolated. Whole-genome genetic analysis of 31 representative strains identified 17 genotypes, reflecting the substantial genetic diversity of LPM-origin H3N8 subtype AIV strains [45].

LPMs serve as critical sites for prolonged viral persistence and multi-subtype co-circulation, providing favorable conditions for continuous genetic reassortment and cross-host transmission of H3 subtype AIV. All reported human H3N8 subtype AIV infection cases have had clear LPM exposure histories, confirming LPMs as key settings for viral spillover from poultry to humans [6,8]. Furthermore, the currently widely recognized novel reassortant variants such as H3N3 were first detected in LPMs, highlighting the important role of LPMs in the emergence and dissemination of novel H3 subtype AIV variants [40,46].

## 3. Genetic Evolution and Reassortment

### 3.1. Genomic Diversity and Evolutionary Rate

H3 subtype AIV continuously accumulates genetic variations through persistent point mutations and high-frequency gene segment reassortment, generating substantial population-level genetic diversity that provides an important genetic basis for cross-host adaptation and epidemic evolution of the virus [20]. Continuous surveillance and whole-genome characterization of H3 subtype AIV in China from 2009 to 2022 demonstrated that, following multiple cross-host introductions from Eurasian wild birds, H3 subtype AIV successfully established and diverged into multiple stable sublineages within domestic duck populations. Over 100 genotypes were identified, among which the H3N2 G23 genotype emerged as the predominant circulating strain in poultry in recent years [26].

H3 subtype AIV may exhibit accelerated evolutionary rates following cross-host transmission, which represents one notable molecular signature in its evolutionary epidemiology. Studies have shown that after wild bird-origin H3 subtype AIVs are introduced into the poultry ecosystem, the nucleotide substitution rate of the HA gene increases significantly, and the evolutionary rate is further elevated within chicken hosts, demonstrating a stepwise acceleration pattern. This characteristic is mainly attributed to the shorter viral generation intervals in poultry hosts and the sustained positive selection pressure imposed by host-adaptive selection [9,26]. This evolutionary pattern reveals the core molecular mechanism of cross-species transmission of H3 subtype AIV: following spillover from wild birds to poultry hosts, shorter generation intervals and host-directed selection jointly drive the continuous acceleration of viral molecular evolution rates [9].

Antigenic characterization of 37 H3 subtype AIV strains isolated from North American wild birds between 2007 and 2011 revealed limited antigenic diversity among the isolates. Hemagglutination inhibition assays showed that all tested H3 avian viruses cross-reacted with each other, with an average antigenic distance of approximately one unit and a maximum of 3.62 units, indicating that these viruses are antigenically relatively stable [47]. Phylogenetic analysis of the HA genes, however, classified these isolates into two genetic clusters, which were distinct from H3 viruses circulating in Eurasian wild birds [47]. These findings suggest that while North-American avian H3 viruses display appreciable genetic diversity, their antigenic diversity remains constrained.

### 3.2. Origin and Evolution of H3N8 Reassortant Viruses

Among all H3 subtype AIVs, the H3N8 subtype exhibits the broadest host tropism and poses a notable zoonotic public health concern. The viruses isolated from human H3N8 cases reported in China between 2022 and 2023 all belong to typical triple-reassortant viruses [6,7,48]. Phylogenetic tracing analysis revealed that the HA genes of these strains belong to the Eurasian lineage, while the NA genes originate from the North American lineage, indicating frequent intercontinental gene reassortment and gene flow among AIVs [49].

The genotype G25 of H3N8 has a unique evolutionary pathway. Whole-genome evolutionary tracing confirmed that the genotype G25 of H3N8 strains are novel reassortant viruses generated through multi-gene segment reassortment involving poultry-origin H3N2 G23 lineage strains, wild bird-origin H3N8 subtype AIV, and locally endemic H9N2 subtype AIV strains [26]. This strain was first detected in chicken flocks in China in July 2021 and subsequently spread rapidly across regions, now having established stable endemic lineages in poultry populations in multiple provinces [27]. Molecular analysis has revealed that the currently circulating H3N8 subtype AIV strains have accumulated multiple key amino acid mutations, which are closely associated with the ability of the virus to bind mammalian receptors and its pathogenicity levels, and the cross-species spillover risk warrants continued monitoring [50,51,52].

From September 2021 to May 2022, nationwide large-scale poultry environmental surveillance yielded 98 H3N8 subtype AIV isolates, of which strains from 10 genotypes carried all six internal gene segments derived from H9N2 subtype AIV [45]. These reassortant strains are primarily concentrated in LPMs and are the direct causative agents of the reported human H3N8 subtype AIV infection cases in China. Of concern, H3N8 subtype AIV exhibits low pathogenicity in poultry, allowing it to circulate covertly in poultry populations for prolonged periods, continuously providing an evolutionary environment for viral gene reassortment, further amplifying the potential public health safety risk [45].

### 3.3. Emergence of Novel H3N3 Reassortant Viruses

In December 2022, a novel chicken-origin H3N3 subtype AIV was isolated in China for the first time; this strain gradually replaced the previously predominant H3N8 subtype AIV and became the new predominant circulating subtype in chicken flocks [20]. Genetic tracing analysis confirmed that this H3N3 subtype AIV is a typical triple-reassortant virus: the HA gene originated from chicken-origin H3N8 subtype AIV, the NA gene originated from H10N3 subtype AIV, and all six internal gene segments were provided by H9N2 subtype AIV [46].

**Figure 2:**
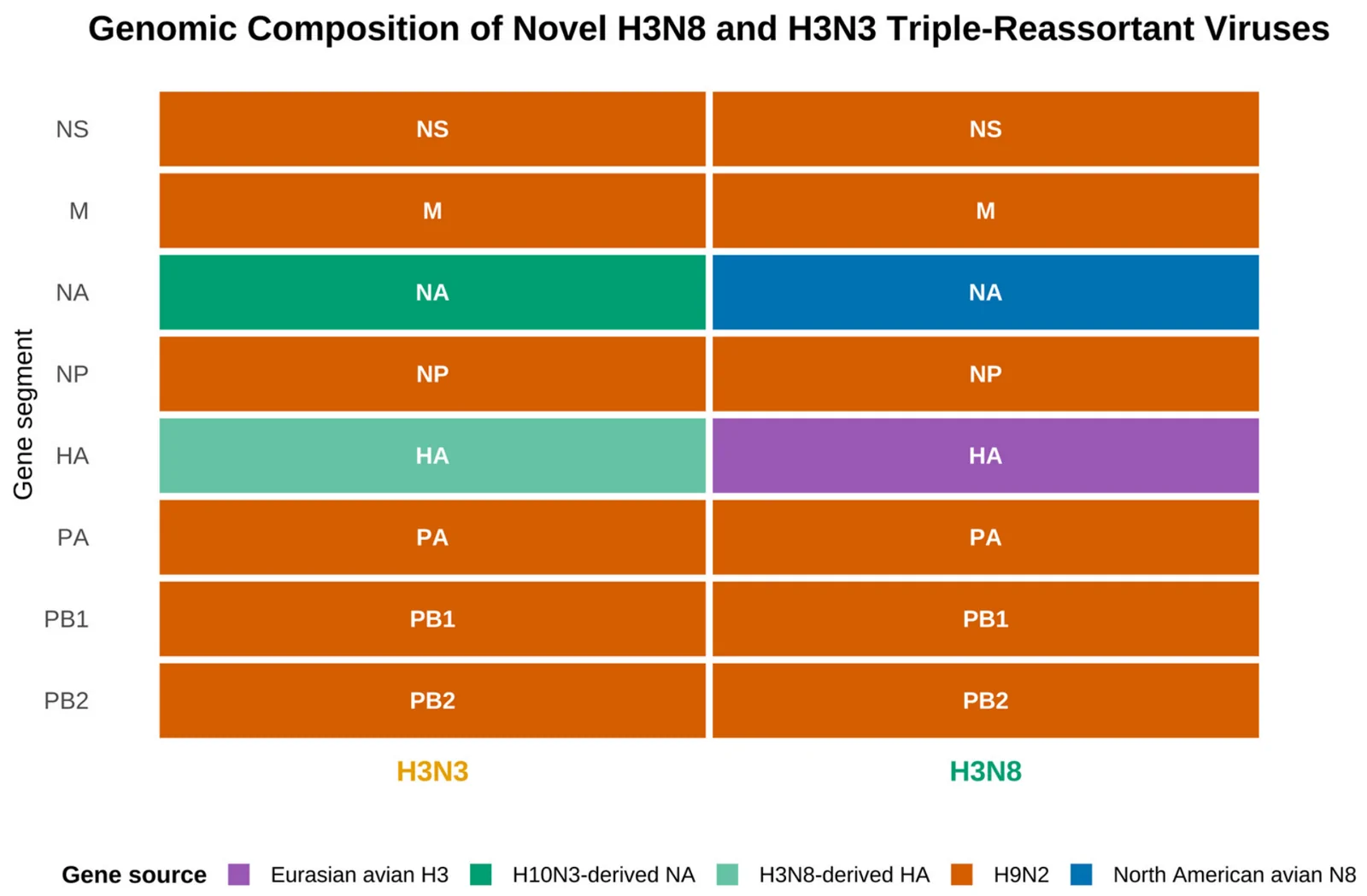
Schematic diagram of the genomic composition of H3N8 and H3N3 triple-reassortant avian influenza viruses. The eight gene segments (PB2, PB1, PA, HA, NP, NA, M, NS) are shown for each virus. Colors indicate the phylogenetic origin of each gene segment: red, H9N2-derived internal genes; purple, Eurasian avian H3; blue, North American avian N8; light green, H3N8-derived HA; dark green, H10N3-derived NA. H3N8 possesses HA from Eurasian avian origin and NA from North American avian origin, with all six internal genes from H9N2. H3N3 acquired its HA from H3N8, NA from H10N3, and all six internal genes from H9N2.

Poultry infection experiments demonstrated that H3N3 subtype AIV can replicate extensively in multiple organs of chickens and pigeons, with experimentally infected animals shedding virus for up to 13 days, and demonstrating efficient horizontal transmission capability within chicken flocks [20]. Receptor binding assays revealed that H3N3 subtype AIV can recognize both avian and human sialic acid receptors, demonstrating prominent cross-host recognition potential [21,46]. Mammalian model experiments further confirmed that most strains can replicate efficiently in mice without causing mortality, while some strains can achieve effective transmission among guinea pigs via respiratory droplets [20]. Collectively, these biological characteristics suggest that the novel H3N3 reassortant virus poses a potential zoonotic spillover risk. The genomic composition of representative H3N8 and H3N3 triple-reassortant viruses is shown in Figure 2.

### 3.4. Other H3 Subtype AIVs

Genetic reassortment of H3 subtype AIV is highly frequent. In addition to the H3N3 and H3N8 subtypes highlighted above, numerous reassortant strains carrying H3 HA segments with different NA combinations have been widely detected in poultry and wild birds, forming a complex and diverse H3NX subtype spectrum [53]. The reassortment process of H3 subtype influenza viruses exhibits significant selection preferences and is not random. Comparative studies have shown that the reassortment frequency of human-origin and swine-origin H3 subtype strains is higher than that of H1 subtype viruses within the same host. Surface glycoproteins of the two major evolutionary lineages of human H1 and H3 subtypes rarely undergo cross-lineage reassortment. This characteristic is attributed to long-term co-evolutionary constraints, where specific pairing of HA and NA proteins significantly enhances viral host adaptability, thereby inhibiting cross-lineage gene reassortment [54].

Among H3 reassortant subtypes, H3N6 subtype AIV is one of the representative subtypes that gained early scientific attention. Surveillance of waterfowl and associated environments in Hunan Province from 2014 to 2015 showed that H3N2, H3N8, H5N6, and other AIV subtypes were co-circulating in the region, giving rise to novel H3N6 subtype AIV reassortant strains. Phylogenetic tracing confirmed that these strains were generated through reassortment between locally circulating H5N6 and other AIV subtypes [25]. The pathogenic risk of H3N1 subtype AIV to poultry is equally also a concern. In 2019, an H3N1 outbreak in Belgian poultry caused severe clinical disease in breeder and layer flocks, resulting in mortality far exceeding expectations [15,16]. H3N2 subtype AIV is the most widely distributed subtype in poultry. From 2022 to 2024, nine H3N2 subtype AIV strains were isolated from domestic ducks in LPMs in Guangxi. Whole-genome characterization revealed that the internal gene origins encompassed H1, H2, H3, H4, H5, H6, H7, H9, and other subtypes, indicating that local H3N2 subtype AIV strains had undergone multiple rounds of complex reassortment. Animal experiments confirmed that three representative strains could stably replicate in the respiratory tissues of both SPF chickens and mice, demonstrating cross-host replication potential [18]. H3N5 subtype AIV also exhibits favorable host adaptability in poultry; goose-origin H3N5 subtype AIV strains can replicate in multiple organs of chickens, with the highest viral loads detected in the cloaca [23]. In addition, surveillance of poultry in Guangdong Province has isolated multiple H3NX subtype AIV strains, and phylogenetic analyses suggest close evolutionary associations with highly pathogenic avian influenza viruses (HPAIVs) [55].

Collectively, the surveillance evidence from these regions demonstrates that H3 subtype AIV continuously generates novel HA-NA combinations through high-frequency genetic reassortment, continuously expanding the genetic diversity of the H3NX spectrum and enriching the pool of viral variants.

## 4. Cross-Species Transmission and Mammalian Adaptation

**Figure 3:**
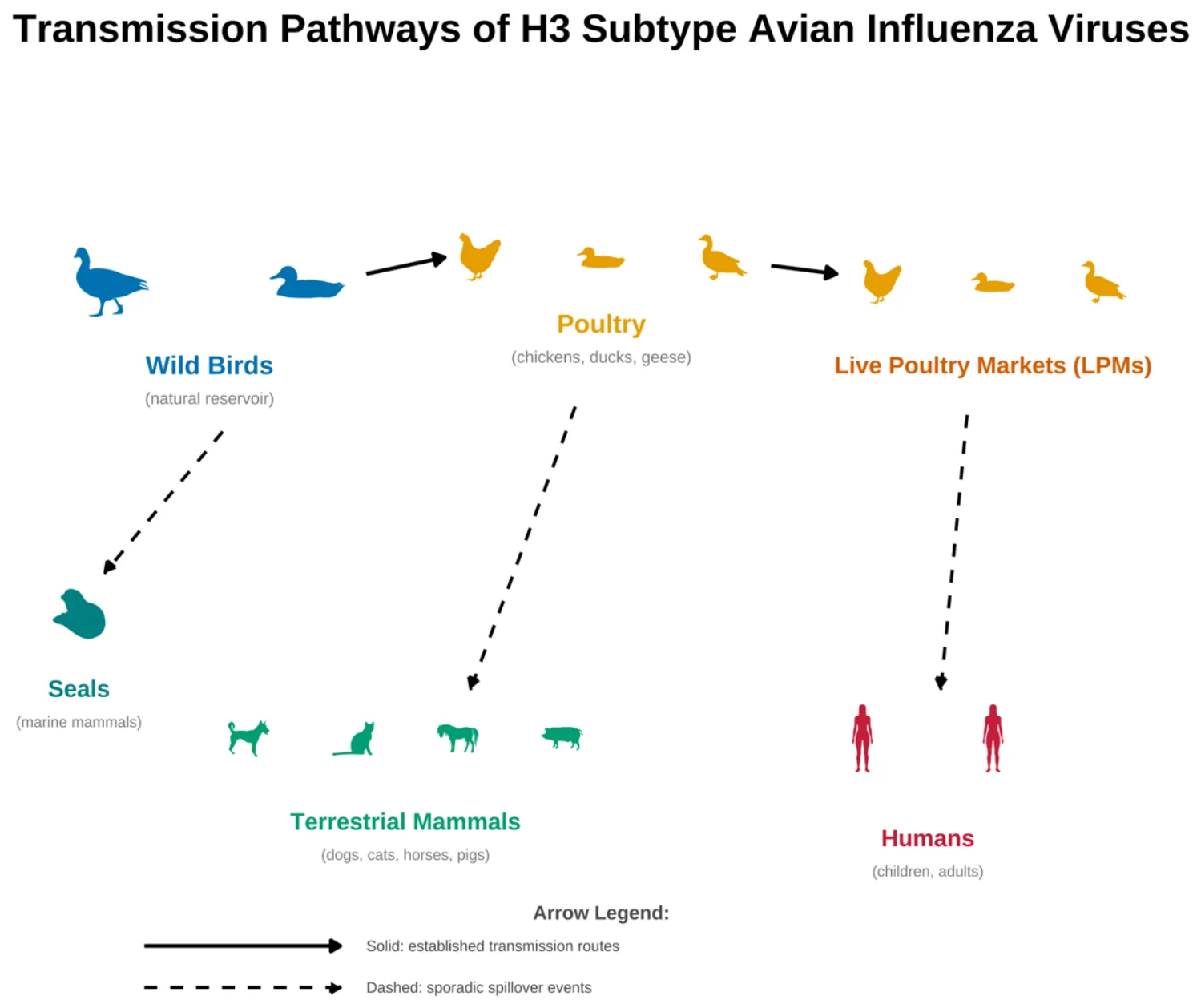
Schematic diagram of the H3 subtype AIV transmission pathways. Wild waterfowl serve as the natural reservoir. The virus transmits from wild birds to poultry and then to live poultry markets (LPMs) via fecal–oral routes. Sporadic spillover events have been documented: from wild birds to seals; from poultry to terrestrial mammals (dogs, cats, horses, pigs); and from LPMs to humans (dashed arrows). Solid arrows indicate established transmission routes; dashed arrows indicate sporadic spillover events. Colors indicate host categories: blue = wild birds, orange = poultry, red = LPMs and humans, teal = seals (marine mammals), green = terrestrial mammals.

The transmission pathways of H3 subtype AIV linking wild birds, poultry, live poultry markets, mammals, and humans are illustrated in Figure 3.

### 4.1. History of Cross-Species Transmission of H3 Subtype AIV

In addition to infecting wild birds and poultry, H3 subtype AIV possesses prominent cross-host transmissibility, with a well-documented history of cross-species spillover. Among these, the H3N8 subtype exhibits the broadest cross-host range, having been detected in dogs, horses, pigs, harbor seals, and humans [7,14,28].

In 1963, equine H3N8 influenza was first reported in the United States and rapidly spread, causing a global pandemic. Phylogenetic tracing and genomic analyses confirmed that the ancestral genes of equine H3N8 influenza viruses originated from wild bird-origin H3 subtype AIV [22].

H3N2 canine influenza virus (CIV) represents a classic example of an avian-origin influenza virus successfully adapting to mammalian hosts. This virus is believed to have crossed from birds to dogs in Asia around 2005 and has since circulated stably in dog populations for nearly two decades [56]; in 2015, it spread beyond Asia for the first time, causing widespread outbreaks in North American dog populations [56]. Phylogenetic trees and Bayesian molecular tracing analyses indicate that H3N2 CIV has now diverged into multiple independent evolutionary lineages [56].

Extensive natural exposure to H3 subtype AIV has been documented in terrestrial wild mammals. Serological surveys of Mongolian wild donkeys revealed that these animals are naturally exposed to H3 subtype AIV, suggesting that wild ungulates represent a long-overlooked natural reservoir host for influenza viruses [57]. Serological screening of backyard poultry in Chile and seroepidemiological surveillance of cynomolgus macaques in Thailand both detected H3 subtype AIV-specific antibodies. These findings further indicate that a wide range of wildlife species in field ecosystems are frequently exposed to and harbor H3 subtype AIV, providing diverse host conditions for viral cross-host dissemination [58,59].

Marine mammals serve as unique and critical hosts for H3 subtype AIV that facilitate cross-species transmission and gene reassortment. Seals, sea otters, and other marine mammals are naturally susceptible to avian-origin influenza viruses and can support co-infection with different viral lineages, with the potential to act as “mixing vessels” for influenza viruses. The representative seal isolate A/seal/Massachusetts/1/2011 (H3N8) not only exhibits substantially enhanced affinity for mammalian-type sialic acid receptors but can also achieve efficient respiratory droplet transmission in ferret models. Such data provide compelling evidence that this strain has overcome avian transmission restrictions and acquired typical mammalian transmissibility [29]. Long-term surveillance of harbor seals along the North American Atlantic coast has shown that H3N8 subtype AIV circulating in the wild can either cause pneumonia and mortality in seals or result in subclinical infections. These outcomes provide a stable environment for long-term viral persistence and accumulation of adaptive mutations [60]. Serological detection of H3 subtype AIV infection in southern sea otters off the California coast further corroborates the broad susceptibility of marine mammals to influenza viruses of diverse origins [61].

### 4.2. Avian-to-Canine Host Jump: Molecular Mechanisms and Public Health Implications

H3N2 CIV represents an invaluable natural model for investigating the molecular mechanisms of influenza virus cross-species host jumps. As a typical avian-origin influenza virus that has rapidly achieved mammalian host adaptation, H3N2 CIV has established stable endemic lineages in global dog populations, with continuous widespread transmission [56]. Contrary to conventional understanding of avian influenza cross-host adaptation, this strain has not acquired the classic mammalian adaptive markers PB2-E627K or D701N during its long-term circulation in dogs. In vitro and animal model experiments have confirmed that artificial introduction of E627K or D701N, although capable of enhancing viral polymerase activity, does not alter the pathogenicity of H3N2 CIV in dogs or mice. Such observations indicate that this strain achieves mammalian host adaptation through a non-classical molecular pathway [56].

The long-term circulation of H3N2 CIV in dogs for nearly two decades offers a unique natural experiment for observing the ongoing adaptation of an avian-origin influenza virus to a mammalian host. Since its emergence around 2005–2007, this virus has established stable endemic lineages in dog populations across Asia and North America, accompanied by progressive antigenic drift and the emergence of novel clades [62,63]. Notably, H3N2 CIV generally lacks the canonical mammalian-adaptive markers PB2-E627K and D701N, which are widely regarded as prerequisites for robust mammalian adaptation [56,63]. Instead, the virus has evolved alternative adaptive pathways driven by cooperative mutations across multiple gene segments. Entropy-based genomic analyses have computationally predicted several NP-associated candidate adaptive signatures (e.g., T373K, A428T, R452K, N473K), although their functional roles remain experimentally unvalidated [62]. The avian-conserved PB2-714S residue has been progressively replaced by 714I among circulating H3N2 CIV isolates; this substitution boosts RNP-complex and overall polymerase activity by improving PB2 nuclear-import efficiency and stabilizing PB2-PA/NP protein–protein interactions [64]. Another functionally verified adaptive change is PA-S184N, which acts synergistically with PB2-E627K to exacerbate pathogenicity in dogs and mice through evasion of host antiviral defenses and induction of potent inflammatory responses [65]. Collectively, these findings illustrate that H3N2 CIV has achieved sustained mammalian fitness via molecular mechanisms distinct from those documented for human-adapted influenza viruses.

The absence of canonical mammalian-adaptive markers in H3N2 CIV challenges the oversimplified paradigm that a fixed suite of mutations is universally obligatory for cross-species host jumps. Rather, influenza A viruses can deploy host-tailored adaptive strategies, exploiting diverse genetic routes to surmount species-specific replication barriers [62]. For instance, the adaptive shift at PB2-714 constitutes one distinct molecular solution enabling efficient mammalian replication, in place of the well-characterized PB2-E627K/D701N substitutions. Furthermore, increasing detection of mutations such as PA-S184N among avian, canine, swine, and human H3N2 viruses suggests step-wise accumulation of adaptive variants as viruses circulate through successive mammalian host populations [65]. This underscores the need for broad surveillance that monitors both canonical and putative non-canonical adaptive markers when assessing the zoonotic risk of emerging H3-variant influenza viruses. A comprehensive appreciation of the full spectrum of host-adaptive molecular signatures is therefore critical for refined risk appraisal and early warning of cross-species spillover events.

The avian-to-canine host adaptation of H3N2 CIV is driven by cooperative mutations at multiple gene sites. The I714S mutation in the PB2 nuclear localization signal region regulates viral nuclear import efficiency and RNP complex assembly, representing a key site for the virus to breach the avian–mammalian host barrier [64]. The V223I substitution in the HA protein reduces binding affinity to human-type SAα2,6-Gal receptors while significantly enhancing viral thermal stability [66]; the HA-W222L mutation further alters viral receptor recognition preference by remodeling the receptor-binding pocket structure, facilitating host adaptive evolution. Long-term surveillance has shown that H3N2 CIV gradually acquires the ability to recognize human-type SAα2,6-Gal receptors during continuous circulation in dogs. Concurrently, HA acid stability in human respiratory epithelial cells is enhanced, indicating a cumulative increase in cross-species spillover risk.

Whole-genome molecular characterization of H3N2 CIV strains isolated in China in 2023 further validated the adaptive pattern of coordinated multi-gene evolution. All eight gene segments of the virus have accumulated amino acid substitutions, among which the mutations PB2-107N, HA1-202I, and M1-227T are consistent with those in currently circulating human H3N2 influenza virus strains [67]. Collectively, these findings imply that the virus continues to optimize its mammalian host adaptation during long-term transmission in dogs, posing a potential zoonotic risk.

### 4.3. Companion Animals as Potential Intermediate Hosts

Dogs and cats, as companion animals in closest contact with humans, have drawn substantial attention regarding their potential intermediate roles in the cross-species transmission chain of influenza A viruses.

H3N2 CIV has established long-term stable epidemic lineages in dog populations in Asia and North America [56]. Phylogenetic tracing analysis of avian-origin H3N2 CIV strains isolated in South Korea from 2009 to 2013 demonstrated that these strains were genetically highly homologous to contemporaneous strains circulating in dog populations in China and South Korea [68]. Animal challenge experiments confirmed that three representative strains could transmit horizontally among dogs via respiratory droplets; compared with the early 2009 isolates, the 2012 strains exhibited significantly enhanced pathogenicity, causing severe clinical symptoms and even mortality in experimental dogs [68]. All strains could successfully infect ferret models, with the 2012 strains reaching higher viral titers in ferret nasal secretions and possessing airborne transmission capability, highlighting prominent cross-species transmission potential [68].

Compared with dogs, cats exhibit higher host susceptibility to H3 subtype AIV. Challenge control experiments revealed that following infection of dogs with H3 subtype AIV strains such as H3N2 and H3N8, only specific serum neutralizing antibodies were induced, with no obvious clinical symptoms observed throughout and no viral shedding or replication detected in tissues [69]. However, cats inoculated with the same H3 subtype AIV strains, although showing no severe clinical disease, seroconverted on days 7, 14, and 21 post-infection, shed virus from nasal secretions for approximately one week, and supported stable viral replication in the lungs, trachea, and nasal turbinates [69]. These differences suggest that cats carry a higher spillover risk in the H3 subtype AIV cross-species transmission chain, highlighting the need for routine influenza pathogen surveillance in domestic cat populations [69].

### 4.4. Mammalian Adaptive Mutations

The ability of H3 subtype AIV to breach the species barrier and establish mammalian host adaptation results from the coordinated accumulation of mutations at multiple gene sites. A single amino acid mutation is insufficient to support efficient viral replication and pathogenicity in mammalian hosts.

The viral polymerase and internal genes contain numerous critical mutation sites that significantly enhance the mammalian adaptation of H3 subtype AIV (Table 2). PB2-E627K emerged as an adaptive mutation during mammalian passage of H3N8 subtype AIV in ferrets, associated with enhanced polymerase activity and transmissibility [27]. PB2-D701N and M1-M192V mutations similarly promote the replication of waterfowl-origin H3N2 subtype AIV in mammalian models and enhance viral pathogenicity [17]. For H3N8 subtype AIV strains, the characteristic mutations PB2-K318R and PB1-F2-N66S upregulate overall viral polymerase activity, simultaneously enhancing virulence and mammalian host adaptation. Wild bird-origin H3N8 subtype AIV strains isolated from Beijing wetland parks have already stably carried these adaptive sites, suggesting that field-circulating strains possess the molecular potential for cross-species spillover [49,50].

H3 subtype AIV strains from different hosts and evolutionary lineages have evolved divergent mammalian adaptive mutation profiles (Table 2). Canine-origin H3N2 CIV achieves efficient colonization in dogs through the unique mutations PB2-292T and PB2-590S [70]. Simultaneously, this strain continuously accumulates amino acid sites such as PB2-107N, HA1-202I, and M1-227T that are highly homologous to those in human seasonal H3N2 influenza viruses. This progressively narrows the molecular adaptation gap between avian-origin viruses and human influenza strains, further amplifying zoonotic transmission risk [67]. The novel reassortant H3N3 subtype AIV, through acquiring PA adaptive mutations carried by the H9N2 backbone genes, significantly enhances polymerase replication efficiency in mammalian cells, providing a genetic advantage for cross-host transmission [46].

Amino acid substitutions and conformational modifications in the HA protein serve as important molecular determinants for H3 subtype AIV to facilitate host switching and achieve immune escape (Table 2). The key substitutions HA-Q226L and HA-G228S can directly reshape the receptor-binding pocket, representing the core molecular basis for the switch in viral receptor preference from avian-type SAα2,3-Gal to human-type SAα2,6-Gal [71]. Beyond classic mutations in the receptor-binding domain, remodeling of HA glycosylation sites represents another important evolutionary strategy for H3 subtype AIV adaptation to mammalian hosts. Multiple sites in the HA antigenic region of H3N2 CIV (D97N, A176T, N204D, V212I, W237L) undergo continuous amino acid substitutions. Such patterns indicate that this strain undergoes persistent antigenic drift during long-term circulation in dogs, allowing it to escape host immune pressure while adapting to the mammalian host environment [70].

In summary, H3 subtype AIV can accumulate mammalian adaptive characteristics through multiple evolutionary pathways, including polymerase functional optimization, internal gene host adaptation, HA receptor recognition preference remodeling, and antigenic drift, laying a comprehensive molecular foundation for efficient viral cross-species spillover and long-term stable evolution in new host populations.

### 4.5. Molecular Basis of Receptor Binding Specificity

The sialic acid receptor binding preference of HA is the core molecular determinant of influenza A virus host restriction [72]. After acquiring cross-species transmission potential, the HA protein of H3N8 subtype AIV gradually evolves the ability to recognize human sialic acid receptors through continuous amino acid mutations [8,71].

Cryo-electron microscopy has revealed, at the molecular level, the structural basis for dual receptor recognition by the H3N8 subtype AIV of human infection origin [71]. Structural analysis found that the HA of this strain possesses dual binding activity for avian and human receptors, with a natural preference for avian-type SAα2,3-Gal receptors. Single-point mutations can significantly remodel viral receptor recognition preference: the Q226L mutation can completely switch viral receptor preference from avian-type to human-type; the G228S substitution both modestly enhances viral binding affinity to human-type receptors and increases overall binding efficiency to both types of sialic acid receptors.

Avian-origin H3N2 subtype AIV also exhibits a dual receptor binding phenotype, capable of recognizing both avian-type SAα2,3-Gal and human-type SAα2,6-Gal sialic acid receptors [73]; some strains in certain evolutionary lineages have accumulated adaptive mutations during long-term circulation, developing a preference for human-type receptor recognition [70]. The novel triple-reassortant H3N3 subtype AIV also displays dual receptor binding characteristics, stably binding to both avian-type and human-type sialic acid receptors [21].

After long-term adaptation in dogs, the receptor binding characteristics of H3N2 subtype AIV HA proteins undergo specific remodeling. H3N2 CIV strains isolated in China in 2025 retain the typical avian receptor preference for SAα2,3-Gal while acquiring moderate binding affinity for human-type SAα2,6-Gal receptors [70]; the HA-V223I substitution carried by these strains, although reducing binding affinity to human-type receptors, significantly enhances HA protein thermal stability, favoring viral survival and transmission in the external environment [66].

A human-isolated H3N8 virus from a fatal case harbors the PB2-E627K substitution, a well-characterized mammalian adaptation marker that enhances polymerase activity in mammalian cells. Solid-phase binding assays demonstrated that this virus is capable of binding human-type sialic acid receptors. In ferret models, this isolate achieved efficient respiratory-droplet transmission even between physically separated animals. Although the original human isolate harbored mixed quasispecies at HA residue 228 (G/S), the HA-G228S variant became enriched during ferret passage. Nevertheless, this substitution alone is insufficient for airborne transmission, whereas PB2-E627K represents the critical molecular determinant [74]. Notably, the HA protein of this virus exhibits acid instability (fusion pH ~5.7–5.8), which may constrain its transmission efficiency relative to human seasonal influenza viruses. These findings provide direct experimental evidence that H3N8 viruses can acquire the capacity for mammalian transmission through adaptive changes in receptor-binding properties and polymerase function.

## 5. Pathogenicity and Immune Responses

### 5.1. Pathogenicity in Avian Species

The classification of avian influenza viruses as low-pathogenicity (LPAI) or high-pathogenicity (HPAI) is defined by the World Organisation for Animal Health (WOAH), based on the intravenous pathogenicity index (IVPI) in chickens or the amino acid sequence at the hemagglutinin cleavage site. This binary classification system was established primarily to differentiate H5 and H7 subtype viruses, in which the presence of a polybasic cleavage site correlates strongly with systemic spread and high mortality. However, the LPAI designation does not preclude the occurrence of severe clinical signs or substantial mortality in specific host–virus–environment contexts. The H3N1 outbreak in Belgium (2019) exemplifies this paradox: although the causative virus retained a monobasic cleavage site and a low IVPI (0.13), it caused up to 60% mortality and 100% egg drop in affected layer flocks [15,16]. This disparity arises because LPAIVs, while lacking the furin-cleavable polybasic cleavage site characteristic of HPAIVs, can acquire virulence through alternative mechanisms. In the Belgian H3N1 strain, the loss of a conserved N-glycosylation site at position 130 of the neuraminidase enabled recruitment of plasminogen for proteolytic cleavage of the hemagglutinin, thereby facilitating systemic spread and neurotropism [16]. Thus, the LPAI/HPAI distinction remains useful for regulatory purposes and for identifying viruses with pandemic potential based on established molecular criteria, but it should not be interpreted as a definitive predictor of clinical outcome across all host species and field conditions.

Traditional H3 subtype AIVs are classified as typical low-pathogenicity avian influenza viruses. Natural infection in poultry predominantly manifests as subclinical infection, causing only mild or even no obvious clinical symptoms, and typically does not result in mass mortality or regional disease outbreaks in poultry [75]. However, in recent years, with the increasing frequency of AIV reassortment events, the biological characteristics of novel reassortant H3 subtype AIVs have undergone significant variation. Compared with traditional strains, their pathogenicity in poultry and transmissibility within populations have both shown marked enhancement.

The currently circulating novel reassortant H3 subtype AIVs in China have exhibited stronger infectivity and enhanced in vivo replication in poultry. Studies have demonstrated that novel H3N8 subtype AIV strains carrying the H9N2 subtype AIV internal gene backbone exhibit significantly superior infectivity and adaptation in chickens compared with traditional H3 subtype strains that have not undergone reassortment [51]. Concurrently circulating novel reassortant H3N3 subtype AIV strains can efficiently colonize the upper respiratory tract and intestinal tissues of chickens, demonstrating efficient in vivo replication and horizontal transmission capacity within chicken flocks [46]. Animal infection experiments further confirmed that the currently circulating H3N3 and H3N8 subtype AIV strains can stably infect SPF chickens and exhibit enhanced pathogenicity. They can effectively transmit and continuously circulate within chicken flocks, distinguishing them from the biological characteristics of traditional low pathogenic strains [53].

### 5.2. Pathogenicity in Mammals

The pathogenicity of H3 subtype AIVs from different evolutionary lineages in mammals exhibits significant inter-strain variation. H3 subtype AIVs isolated in China from 2021 to 2022 displayed only low pathogenicity in mouse infection models [51]. In contrast, the novel reassortant strains generated through genetic reassortment showed significantly enhanced mammalian host adaptation and in vivo pathogenicity. Novel triple-reassortant H3N3 subtype AIV strains can efficiently proliferate in mouse lung tissues and nasal turbinates, causing body weight loss exceeding 10% in infected mice, with markedly enhanced pathogenicity [21]. Both novel triple-reassortant H3N3 and H3N8 subtype AIVs can effectively infect BALB/c mice and Hartley guinea pigs, and H3N8 subtype AIV can achieve efficient horizontal transmission among guinea pigs through direct contact [53]. Novel H3N2 subtype AIV reassortant strains can replicate efficiently in mice and exhibit strong pathogenicity, demonstrating prominent cross-species pathogenic potential [18].

The pathogenicity of H3 subtype AIV in mammals is not regulated by a single gene independently, but results from the synergistic effects of multiple gene segments. A previous study by our group demonstrated that the enhanced mammalian pathogenicity of H3N2 subtype AIV has a defined molecular basis. Using a wild bird-origin H3N2 subtype AIV as the study subject, a mammalian-adapted strain was successfully generated through serial passaging in BALB/c mice. The adapted strain exhibited significantly enhanced pathogenicity and replication capacity in mice, as evidenced by a reduced mouse median lethal dose (MLD_50_), exacerbated body weight loss, and the ability to replicate in multiple organs (lung, nasal turbinates, spleen, kidney, and brain). Whole-genome sequencing revealed that the adapted strain accumulated eight amino acid substitutions in the PB2, PB1, HA, NA, and M genes: PB2-E192K, PB2-D701N, PB1-F269S, PB1-I475V, PB1-L598P, HA-V242E, NA-G170R, and M1-M192V. Four of these substitutions have been identified in naturally occurring isolates, suggesting that wild bird-origin H3N2 subtype AIV poses a potential mammalian adaptation risk. Two critical molecular markers regulating mammalian pathogenicity were further identified: PB2-D701N and M1-M192V [17,76]. Another study on H3N8 subtype AIV similarly confirmed the critical role of PB2-D701N: following serial passaging of wild bird-origin H3N8 subtype AIV in mice, adapted strains exhibited significantly enhanced virulence in mammals. Gene sequencing revealed that all adapted strains stably carried PA-T97I, whereas one strain additionally acquired the PB2-D701N mutation [77]. These studies collectively confirm the critical role of the PB2-D701N mutation in enhancing the mammalian pathogenicity of H3 subtype AIV, providing important molecular targets for cross-species transmission surveillance and risk assessment of H3 subtype AIV.

The pathogenic potential of human-isolated H3N8 viruses has been systematically evaluated in mammalian models. A virus isolated from a fatal human case exhibited significantly enhanced virulence in mice, with a median lethal dose (MLD_50_) of 10^5.5^ TCID_50_, compared with chicken-origin isolates, which caused no mortality at equivalent challenge doses. In ferrets, this human isolate provoked severe clinical signs including wheezing, dyspnea, and coughing; viral replication was detectable in multiple respiratory tissues as well as the brain. By contrast, chicken-origin isolates induced only mild infection largely restricted to the upper respiratory tract. In human respiratory epithelial cell systems, the virus replicated efficiently in both normal bronchial epithelial cells and lung epithelial cells, producing viral titers 100- to 1000-fold higher than chicken-derived H3N8 isolates. Notably, serum specimens collected from recipients vaccinated with seasonal H3N2 influenza vaccines lacked cross-reactive neutralizing activity against this H3N8 virus, indicating that human populations remain largely immunologically naive to this emerging mammalian-adapted virus [74].

### 5.3. Immune Responses and Host–Pathogen Interactions

#### 5.3.1. Innate Immune Recognition

H3N1 LPAIV elicits a robust yet delayed innate immune response in chicken oviduct organ cultures (OOCs) compared with H9N2. At 48 h post-infection, H3N1 induces an 8-fold elevation in IFN-λ mRNA and a 256-fold upregulation of CCL4, correlating with markedly higher viral loads and more severe histopathological lesions, including extensive cilia loss, epithelial exfoliation, and injury to the lamina propria [78]. By contrast, H9N2 triggers earlier but milder responses, with significant induction of antiviral genes such as CCL4, IFIT5, MX1, and LYZ [78].

Host genetic background modulates the magnitude of innate immune responses to H3N1 and H6N1 infection. Brown layer (BL)-derived OOCs show lower basal IFN-λ mRNA levels than white layer (WL) OOCs, yet exhibit greater IFN-λ induction upon H3N1 and H6N1 challenge. WL OOCs are less permissive to H6N1 infection, with reduced viral loads and weaker IFN-λ responses [78].

Transcriptomic profiling of H3N1-infected BL OOCs reveals a distinct innate immune signature characterized by pronounced upregulation of CCL5, PPARG, IL6, and TLR7 at 48 h post-infection. Both H3N1 and H9N2 induce multiple interferon-stimulated genes (ISGs), including IFIT5, IFITM3, IFITM5, OASL, and ISG12-2, whereas transcripts encoding type I IFNs (IFN-α and IFN-β) remain largely unaltered, suggesting a limited role of type I IFN in restricting LPAIV within reproductive-tract tissues [78].

#### 5.3.2. Host-Species-Specific Immune Responses

Immune responses following H3-subtype AIV infection vary substantially across avian hosts. In chickens, H3N8 triggers the fewest differentially expressed genes (DEGs) among the LPAIV subtypes tested, with 32% of DEGs being colon-specific. In contrast, tufted ducks exhibit the largest DEG repertoire, with 51% of DEGs exclusive to colon tissues [79]. This stark contrast suggests that chickens mount relatively constrained transcriptional responses to H3N8, whereas tufted ducks launch more potent host-defense programs [79].

Despite the presence of a functional RIG-I gene in tufted ducks, its transcript abundance is neither elevated nor repressed upon H3N8 infection [79]. Together with the generally weak or undetectable induction of interferon-related genes in both species, this finding implies that LPAIVs such as H3N8 may evade efficient innate immune sensing or actively suppress host immune cascades. Indeed, H3N8 and H4N6 infections yield fewer DEGs in chickens relative to other AIV subtypes (e.g., H6N2), indicating that certain viral lineages possess superior capacity to circumvent or remodel host immune signaling [79].

Gene ontology enrichment for DEGs from H3N8-infected chickens and tufted ducks identifies limited enrichment for immune-system-process genes (GO:0002376). Notable exceptions include chicken gamma-glutamyltransferase 2, as well as tufted-duck colony-stimulating factor 1 receptor and interleukin-16 [79]. These data underscore profound inter-species heterogeneity in host responses to H3N8. Host-intrinsic factors, including RIG-I functionality and divergent PRR-signaling circuits, are therefore proposed to drive disparate infection outcomes of H3-subtype AIV among avian species [79].

#### 5.3.3. NS1-Mediated Immune Evasion

Influenza A virus non-structural protein 1 (NS1) acts as a major antagonist of host interferon signaling and serves as a central effector for immune evasion and host adaptation. Compared with H5N1 and H9N2, immune-evasion mechanisms deployed by H3-subtype AIV remain under-characterized. Recent investigations into seal-origin H3N8 viruses nevertheless offer valuable insights into NS1 functional evolution following cross-species spillover [80].

Seal-adapted NS1 exhibits comparable expression levels and interferon-antagonistic activity to its avian ancestor. However, discrete amino-acid substitutions within the NS1 effector domain (notably residues 94, 104 and 171) reshape NS1 functionalities: they enhance protein stability, blunt interferon induction, trigger host transcriptional shut-off, and augment viral polymerase activity in human cells, without affecting NS1 expression or viral replication in avian cells [80]. These findings illustrate that NS1 undergoes host-adaptive functional evolution upon avian-to-mammal transmission and provide mechanistic clues for influenza A virus adaptation to mammalian hosts [80].

In chickens, H3N1 and H6N1 NS1 harbor residue 96E, which preserves NS1-dependent binding and inhibition of TRIM25—a critical interferon activator. This molecular feature may partly explain the marginal type I IFN mRNA induction observed during H3N1 infection [78]. Furthermore, the D171Y substitution within H9N2 NS1 (absent in H3N1 and H6N1) markedly dampens IFN-λ and pro-inflammatory cytokine production, correlating with lower IFN-λ transcript abundance in H9N2-infected OOCs compared with H3N1-challenged OOCs [78]. Collectively, strain-specific NS1 polymorphisms likely underpin divergent innate immune responses triggered by distinct H3-subtype AIV isolates.

While NS1-driven immune-evasion strategies of H3-subtype AIV in avian hosts warrant further experimental validation, existing evidence supports multiple immunomodulatory strategies deployed by H3 viruses: (i) NS1-mediated interferon antagonism; (ii) repression of type I IFN responses; and (iii) remodelling of host transcriptomes via NS1 effector-domain functions. Conservation of these mechanisms across H3 strains and host taxa reinforces NS1 as a pivotal virulence determinant and a promising target for antiviral therapeutic development.

#### 5.3.4. Adaptive Immune Responses

Vaccine studies have yielded key insights into adaptive immunity elicited by H3-subtype AIV. A recombinant adenovirus-5-vectored vaccine expressing H3N8 hemagglutinin (rAd-HA) induces robust humoral and cellular immune responses in both mice and chickens. Hemagglutination-inhibition (HI) antibody titres reach ≥64 in animals immunized via either intramuscular or intranasal routes, and intranasal delivery efficiently stimulates secretory IgA (sIgA) production within the respiratory mucosa [81]. Vaccinated mice achieve 100% survival against lethal H3N8 challenge, accompanied by effective suppression of pulmonary viral replication. In SPF chickens, rAd-HA vaccination fully abolishes viral shedding in oropharyngeal and cloacal swabs upon H3N8 challenge, highlighting its potential to block viral transmission [81].

Cytokine profiling reveals that rAd-HA vaccination significantly upregulates pulmonary IFN-γ mRNA in mice, whereas pro-inflammatory mediators (TNF-α, IL-6) and Th2-associated cytokines (IL-4, IL-13) remain largely unmodified [81]. Such Th1-biased immunity, characterized by elevated IFN-γ without excessive inflammation or Th2-skewing, presumably facilitates efficient viral clearance while minimizing immunopathology [81].

An oil-emulsion-inactivated vaccine based on a novel chicken-origin H3N3 isolate (A/chicken/China/YC01/2023(H3N3)) also induces high-level antibody responses in SPF chickens, with HI antibody titres peaking at 9.6 log_2_ four weeks post-vaccination [82]. Upon homologous or heterologous duck-origin H3N3 challenge, vaccinated chickens display no clinical signs, and viral shedding is undetectable by day 4 post-challenge, confirming cross-protection against antigenically related H3N3 strains [82].

Taken together, these findings confirm that vaccine-elicited adaptive immunity can confer effective protection against H3-subtype AIV. Successful induction of both systemic immunity (HI antibodies, IFN-γ-producing T-cells) and mucosal immunity (sIgA) suggests that multiple immunization regimens, including intramuscular and intranasal administration, can be exploited to develop poultry-targeted H3-AIV vaccines, with prospective applicability for human populations.

#### 5.3.5. Knowledge Gaps and Future Directions

Despite recent research progress, critical knowledge gaps persist regarding immune signaling upon H3-subtype AIV infection and host–pathogen interaction mechanisms.

First, the molecular underpinnings of H3-subtype AIV innate immune evasion—especially NS1-driven interferon antagonism—remain incompletely defined in avian hosts. Most functional studies of H3 virus NS1 have concentrated on strains isolated from mammalian hosts, including seals, and have been carried out mainly in cell culture systems [80]. The extent to which these findings can be translated into avian-relevant contexts remains poorly understood, given that birds represent the natural reservoirs and primary hosts for H3 AIV. Systematic characterization of NS1 mutations in avian-origin H3 isolates and their impacts on IFN-related cascades (including RIG-I/MDA5, JAK-STAT and NF-κB pathways) is urgently required [78,80].

Second, the functional role of RIG-I during H3-subtype AIV infection in ducks and other non-chicken avian species merits further investigation. Although tufted ducks encode functional RIG-I, its transcript level is not perturbed during H3N8 infection [79]. It remains unresolved whether RIG-I shapes inter-species variation in host susceptibility to H3-subtype AIV, and whether H3 viruses have evolved countermeasures to neutralize RIG-I-mediated antiviral defense.

Third, although vaccine-induced adaptive immunity has been well documented for H3N8 and H3N3 [81,82], immune correlates of protection await further dissection. In particular, the relative contributions of neutralizing antibodies, mucosal sIgA and T-cell responses remain to be quantified. Additionally, durability of vaccine-induced protection and risks of vaccine escape driven by antigenic drift among circulating H3 strains represent major considerations for long-term H3-AIV control strategies.

## 6. Detection, Surveillance, and Prevention

### 6.1. Detection Methods

Accurate and rapid detection of H3 subtype AIV is essential for epidemic surveillance, source tracing, and early warning, and is critically important for blocking cross-host transmission and reducing public health risks. A diversified technical system encompassing molecular detection, immunological detection, and isothermal amplification has been gradually established for H3 subtype AIV, meeting diverse application needs, including precise laboratory identification, large-scale screening, and rapid on-site diagnosis at primary levels.

#### 6.1.1. Molecular Detection Techniques

Molecular detection, characterized by high specificity and sensitivity, has become the mainstream technology for H3 subtype AIV identification and subtyping. Multiplex reverse transcription PCR (RT-PCR) enables simultaneous identification of multiple subtypes, significantly improving detection throughput and screening efficiency. A multiplex RT-PCR system based on the GeXP analyzer can simultaneously detect eight zoonotic avian influenza subtypes, including H3, making it suitable for identification of mixed infections and large-scale epidemiological surveillance [83]. A triplex real-time PCR system can simultaneously identify three high-risk avian influenza subtypes, including H3, and is suitable for routine surveillance in poultry farms and LPMs [84].

#### 6.1.2. Immunological Detection Techniques

Immunological detection methods are simple to perform and require minimal equipment, making them valuable tools for rapid preliminary screening at the primary level. A quantum dot-based immunochromatographic strip can specifically recognize the HA1 protein of H3 subtype AIV, requiring only 15 min per assay with a visual detection limit of 15.63 ng/mL. This method combines speed and sensitivity, and is suitable for immediate qualitative detection on farms and in field settings [85]. A double-antibody sandwich ELISA using specific monoclonal antibodies offers strong specificity, high sensitivity, and good reproducibility for H3 subtype AIV antigen detection, enabling high-throughput quantitative testing and serving as a core method for routine laboratory surveillance and seroepidemiological surveys [86].

#### 6.1.3. Isothermal Amplification Rapid Detection Techniques

Isothermal amplification techniques eliminate the need for temperature-cycling equipment required by traditional PCR, making them more suitable for resource-limited settings and on-site rapid testing. An RT-RAA-based rapid detection method for H3 subtype AIV can efficiently amplify nucleic acids under isothermal conditions, with products visualized using portable blue light devices. This method offers simple operation with lightweight equipment, making it suitable for primary surveillance stations and field emergency screening [87]. Additionally, a CRISPR-Cas13a-based isothermal detection system coupled with lateral-flow strips has been applied for rapid avian influenza detection. This platform features high specificity and rapid visual readout, making it suitable for field-deployable diagnostics. Although the reported assay was optimized exclusively for H5 subtype detection and has not yet been validated for H3 viruses, the inherent flexibility of this platform—via redesigning subtype-specific CRISPR RNAs—suggests substantial potential for adaptation to H3 detection. Future development of H3-specific CRISPR-Cas13a assays would constitute a valuable addition to the on-site diagnostic toolkit for H3 subtype AIV surveillance [88]. The key performance characteristics of H3 subtype AIV diagnostic methods are summarized and compared in Table 3.

### 6.2. Global Surveillance Networks

In recent years, evidence indicates broadening host spectrum and enhanced cross-host adaptive capacity among some H3 subtype AIV variants, raising concerns regarding their zoonotic spillover potential for global public health security. Establishing a multi-dimensional, routine, and cross-regional integrated global surveillance network is essential for accurately tracking H3 subtype AIV epidemiological dynamics, tracing viral evolution, and providing early warning of cross-species transmission risks [89]. Current routine surveillance for H3 subtype AIV is built around three core scenarios: LPMs, wild birds, and mammals. Through continuous, comprehensive dynamic surveillance, it enables early identification and preemptive intervention of viral variation, genetic reassortment, and cross-host transmission risks.

#### 6.2.1. Live Poultry Market Surveillance

LPMs, as core venues for poultry trading, aggregation, and mixed rearing, provide favorable conditions for continuous viral aggregation, proliferation, and genetic recombination. They represent high-risk nodes for avian influenza transmission and evolution, and constitute the core frontline for epidemiological surveillance [90]. Environmental surveillance data from Chinese LPMs (2019–2023) showed that, compared with highly pathogenic avian influenza, detection rates of low-pathogenicity avian influenza viruses, including H3, have increased year by year, with increasingly prominent covert circulation characteristics [43]. Low-pathogenicity H3 infection in poultry presents no typical clinical symptoms, is highly covert, and can easily be missed by routine screening. The virus circulates continuously in poultry populations and the environment, accumulating adaptive mutations and substantially increasing the probability of generating novel reassortant strains. Therefore, routine environmental sampling and pathogen surveillance in LPMs are key measures for early detection of H3 variant dynamics and preemptive mitigation of potential outbreak risks [34].

#### 6.2.2. Wild Bird Surveillance

Wild waterfowl serve as the natural reservoir hosts and primary transmission vectors for H3 subtype AIV, with the virus capable of long-term circulation and evolution in wild bird populations, representing an important source driving global intercontinental spread of this subtype [10]. Long-term surveillance of wild birds in South Korea, Mongolia, and Kazakhstan has confirmed that H3 is one of the most frequently detected subtypes in migratory waterfowl, reflecting its widespread prevalence in Eurasian wild birds [34,38,39]. Seasonal migration of wild birds represents the core pathway for transboundary viral spread, and year-round uninterrupted surveillance along migratory routes plays an important supporting role in elucidating H3 transmission pathways and analyzing genetic evolution patterns.

A year-round surveillance study of mallards on Lake Erie, Ohio, USA, demonstrated that traditional autumn and winter concentrated sampling has significant blind spots. Year-round continuous surveillance can capture viral evolutionary characteristics during under-sampled periods such as spring, compensating for data gaps from seasonal sampling and improving understanding of annual H3 prevalence patterns [91]. Beyond temporal coverage, targeted spatial surveillance is equally critical. The Delmarva Peninsula, USA, located at the intersection of the Atlantic migratory bird stopover site and large-scale poultry production areas, has long-term surveillance demonstrating that overlap zones between migratory corridors and intensive poultry production areas are high-risk regions for cross-host viral spillover. Targeted surveillance deployment in these areas can effectively enhance early warning capacity for cross-species transmission [92].

#### 6.2.3. Mammalian Surveillance

Existing studies have confirmed that H3 subtype AIV possesses broad mammalian infectivity, capable of breaching species barriers to infect multiple terrestrial and marine mammals, with a host range far exceeding avian species [57]. Therefore, incorporating mammals into routine surveillance systems is a key step to address prevention and control gaps and improve the comprehensive surveillance network. Pigs, as classic influenza “mixing vessels,” can support co-infection and genetic reassortment of avian, human, and mammalian influenza viruses simultaneously, readily generating novel H3 subtype reassortant strains adapted to mammals, playing a significant role in viral cross-species adaptive evolution [93].

Pigs are recognized as classic influenza “mixing vessels” because they express both avian-type (SAα2,3-Gal) and human-type (SAα2,6-Gal) sialic acid receptors in their respiratory tracts, rendering them susceptible to co-infection with avian, human, and swine influenza viruses. Such co-infections provide opportunities for genetic reassortment, potentially generating novel H3 subtype reassortants with enhanced mammalian adaptation and zoonotic potential. Notably, direct virological evidence has demonstrated that pigs can be naturally infected with avian-origin H3 subtype influenza viruses; an avian-like H3N2 swine influenza virus was isolated from pigs with severe respiratory disease in southern China, with all eight gene segments originating from domestic aquatic birds [19]. In addition, experimental infection studies have confirmed that H3N8 AIV can replicate efficiently in pigs, and serological evidence indicates natural exposure of pigs to H3 subtype viruses in some regions [28,93]. These findings underscore the need for systematic surveillance of swine populations as an integral component of H3 AIV monitoring.

Despite this recognized risk, routine surveillance for H3 subtype AIV in swine remains fragmented and geographically uneven, representing a significant gap in the current early warning system. Establishing systematic, longitudinal surveillance programs in swine populations, particularly in regions with intensive pig production and proximity to poultry operations, would enable early detection of H3 virus incursions into swine and timely identification of novel reassortants with pandemic potential. Integration of swine surveillance into the proposed multi-host monitoring framework under the One Health approach would therefore strengthen cross-sectoral coordination and enhance preparedness against emerging H3 subtype AIV variants.

Wildlife surveillance in Europe has shown serological evidence of exposure to multiple influenza virus subtypes including H3 in wild boar populations in Spain, indicating widespread covert circulation and cross-host transmission risk of H3 subtype influenza viruses in wild mammals [94]. This demonstrates that avian-only surveillance has significant limitations. Establishing an integrated surveillance system covering poultry, livestock, and wild mammals can promptly detect cross-species transmission signals of H3, which is of substantial practical importance for reducing zoonotic risks and strengthening public health defenses. The three surveillance nodes—LPMs, wild birds, and mammals—complement each other and share data, forming a complete global H3 avian influenza early warning network that provides continuous and comprehensive epidemiological support for outbreak response, viral tracing, and vaccine development.

### 6.3. Vaccine Development

Compared with highly pathogenic avian influenza subtypes, H3 subtype AIV has historically received insufficient attention due to its low pathogenicity in poultry and lack of typical clinical manifestations, resulting in limited vaccine research investment and a lack of dedicated vaccine systems. However, in recent years, multiple reports of cross-species human infections with H3N3 and H3N8 subtype AIVs from various regions worldwide have indicated that H3 subtype AIV possesses zoonotic transmission potential. The public health risk of novel variant strains warrants continued attention, making the iterative development and technological innovation of H3 avian influenza vaccines an urgent task in current avian influenza prevention and control. Currently, H3 subtype AIV vaccine development has advanced across multiple technical platforms, including traditional inactivated vaccines, vector vaccines, and mRNA vaccines, providing a solid foundation for precise prevention and broad-spectrum early warning of H3 avian influenza.

A critical practical challenge in H3 subtype AIV vaccination is the differentiation of infected from vaccinated animals (DIVA) via routine serological testing. Conventional inactivated vaccines do not allow serological distinction between vaccinated and naturally infected birds, complicating surveillance and outbreak response. To address this limitation, the development of marker vaccines (e.g., marker vaccines based on modified-NA or variant-HA antigens) paired with companion diagnostic tests represents a promising direction. Such DIVA-compatible strategies would enable continued serosurveillance even in vaccinated populations, facilitating timely detection of field virus circulation and supporting evidence-based control decisions. Future research should prioritize the development of DIVA-compatible H3 vaccine candidates to maximize the utility of vaccination programs in poultry.

A further consideration for H3 subtype AIV vaccination is the challenge of selecting appropriate vaccine strains in the face of ongoing antigenic drift. This is not a problem unique to H3—experience with H5 and H7 avian influenza control has shown that field viruses can diverge antigenically from vaccine strains over time, necessitating periodic seed strain updates. Several potential approaches could be considered to address this challenge for H3 viruses. First, enhanced antigenic surveillance of circulating field isolates, paired with routine hemagglutination inhibition testing against reference sera, would facilitate early detection of emerging antigenic variants. Second, existing collaborative frameworks such as the OFFLU Avian Influenza Matching (AIM) initiative, which currently provides antigenic characterization data to support H5 vaccination programs, could potentially be extended to include H3 subtype viruses, as similar standardized approaches have been proposed for other subtypes. Third, mRNA-based vaccine platforms offer faster manufacturing timelines and may allow strain selection to occur closer to the time of vaccine deployment, potentially improving antigenic match. These are offered as potential directions for consideration rather than as established solutions; the inherent challenge of antigenic drift in H3 viruses will likely require ongoing surveillance and iterative adaptation of vaccination strategies.

#### 6.3.1. Traditional Poultry Vaccines

Traditional inactivated vaccines represent the mainstream vaccine type for avian influenza prevention and control, characterized by high safety and stable protection, suitable for large-scale poultry immunization. The CK/NT308/H3N3 strain is a promising vaccine candidate. The inactivated vaccine prepared from this strain provides complete clinical protection against both H3N3 and H3N8 epidemic strains and effectively blocks viral shedding in infected poultry, thereby interrupting the transmission chain and holding promise for field application [23]. Furthermore, a novel chimeric H3N2 candidate vaccine based on an MDCK suspension cell culture platform can elicit efficient specific immune responses in mice, stably inducing hemagglutination-inhibiting, neutralizing, and specific IgG antibodies, while also stimulating mucosal IgA antibodies in bronchoalveolar lavage fluid. This achieves dual humoral and mucosal immunity, providing a new technical approach for industrial production of cell-based H3 avian influenza vaccines [95].

#### 6.3.2. Adenovirus-Vectored Vaccines

Vector vaccines offer advantages including strong immunogenicity, flexible administration routes, and short development cycles, representing an important direction for novel H3 subtype AIV vaccine research. A human adenovirus type 5-vectored H3N8-HA recombinant vaccine (rAd-HA) induced robust humoral and cellular immune responses in BALB/c mice and SPF chickens. Following intramuscular injection or intranasal administration, hemagglutination-inhibiting antibody titers in animals reached ≥1:64, with simultaneous upregulation of IFN-γ and other cellular immune factors. Intranasal immunization effectively stimulated mucosal secretory IgA production in the respiratory tract, establishing a mucosal immune barrier [81]. Challenge protection experiments confirmed that this vaccine fully protected mice against lethal H3N8 subtype AIV challenge. It effectively inhibited viral replication and alleviated pulmonary histopathological damage, providing important technical support for emergency prevention and control of H3N8 subtype AIV [81].

#### 6.3.3. mRNA Vaccines

mRNA vaccines, with advantages including rapid development, high safety, and adaptability to variant iteration, have become a cutting-edge technology for prevention and control of emerging influenza variants. An H3N8 HA mRNA-LNP vaccine prepared using a lipid nanoparticle (LNP) delivery system can efficiently express H3N8 subtype AIV hemagglutinin antigen. Mouse immunization showed that two doses of intramuscular immunization induced high-level specific humoral immune responses [96]. Subsequent challenge experiments confirmed that this vaccine significantly alleviated body weight loss, accelerated recovery, completely cleared viral loads in lung tissues, and effectively alleviated pulmonary inflammation and pathological damage. These results demonstrate excellent immune protection against H3N8 subtype AIV, providing a novel technological pathway for rapid development and emergency stockpiling of H3 subtype vaccines [96].

### 6.4. Prevention and Control Challenges

The unique epidemiological and evolutionary characteristics of H3 subtype AIV, coupled with cross-host transmission features and existing gaps in current disease prevention and control systems, create multiple practical bottlenecks that continuously pose public health challenges. Based on epidemiological surveillance data and global research findings, the prevention and control challenges for H3 subtype AIV are concentrated in five aspects: covert viral circulation, inadequate surveillance systems, frequent genetic reassortment, prominent cross-species spillover risks, and insufficient subtyping diagnostic capacity at primary laboratories.

Although H3 subtype AIV is classified as LPAIV, certain field outbreaks can cause substantial economic losses. The H3N1 outbreak in Belgium (2019) affected 82 poultry holdings within 16 weeks and resulted in an up-to-100% drop in egg production and 60% mortality in affected layer flocks, with recovered animals never fully regaining their laying potential, leading to sustained production losses [15,16]. These losses were severe enough to prompt restriction measures normally reserved for highly pathogenic avian influenza outbreaks [16]. Although no such data are yet available for H3-subtype viruses, experiences with other LPAIVs (e.g., H7N9) suggest that human spillover events could also trigger consumer-driven market disruptions and temporary live-poultry-market closures, and pose a plausible indirect economic risk for H3-subtype viruses [75].

First, the low pathogenicity of the virus results in covert transmission, making field detection and identification difficult. Most H3 subtype AIVs belong to LPAIVs; natural infection in poultry presents no typical characteristic clinical signs, manifesting only mild subclinical infections without causing large-scale poultry mortality, making them easily overlooked in routine livestock epidemic prevention inspections. The virus can circulate covertly for extended periods in poultry farming environments, LPMs, and poultry populations, providing ample temporal windows for the accumulation of adaptive mutations and multi-subtype genetic reassortment [26].

Second, current surveillance systems have structural gaps, resulting in weak preemptive early warning and control capabilities. At present, routine active surveillance coverage for H3 subtype AIV is limited, and sampling frequency is insufficient. There is also a lack of targeted prevention and control interventions. Consequently, it is difficult to accurately predict viral epidemiological dynamics and genetic variation trends, and prevention and control efforts often lag behind viral evolutionary processes [11].

Third, frequent genetic reassortment continuously increases the complexity of comprehensive prevention and control. H3 subtype AIV can co-circulate and co-infect with multiple avian influenza subtypes, including H9, continuously undergoing multi-segment gene exchange. This generates novel reassortant strains with entirely new genetic backgrounds and unknown biological phenotypes. Continuous antigenic drift reduces the protective efficacy of existing vaccines, substantially increasing the difficulty of outbreak management and zoonotic risk warning [97].

Fourth, cross-host colonization and cross-species spillover risks remain prominent. H3 subtype AIV has already established stable adapted lineages in multiple mammalian species including dogs and horses. Mammalian H3 subtype influenza viruses such as canine H3N2 and equine H3N8 can persist and circulate independently for prolonged periods, constituting persistent animal viral reservoirs, posing significant safety risks of repeated cross-species spillover and infection in humans [68].

In addition, insufficient subtyping diagnostic capacity at primary laboratories further constrains the effectiveness of comprehensive prevention and control. Proficiency testing for animal influenza diagnostics conducted in the Netherlands in 2023 revealed that all 50 participating primary clinical laboratories could stably detect influenza A viruses, but most lacked comprehensive subtyping diagnostic systems, making it difficult to accurately differentiate animal-origin influenza virus subtypes including H3 [98]. This finding suggests that animal influenza virus subtype-specific diagnostic capacity is generally deficient worldwide, and there is an urgent need to strengthen subtyping diagnostic technical capabilities at primary laboratories and improve the precise diagnosis and early warning system for H3 subtype AIV.

## 7. Discussion and Prospects

H3 subtype AIV has long been excluded from priority avian influenza prevention and control efforts due to its low pathogenicity, and its public health risk has been generally underestimated. However, in recent years, accumulating observations of enhanced cross-host adaptive capacity and more frequent genetic reassortment events have gradually repositioned H3 subtype AIV from a neglected low-pathogenic avian pathogen to an influenza subtype of concern owing to its zoonotic spillover and pandemic-related risks. Multiple human H3N8 subtype AIV cases reported in China from 2022 to 2023 confirmed that H3 subtype AIV can effectively breach the species barrier and cause natural cross-species infections, marking the transition of its public health threat from a theoretical risk to a realistic concern.

The studies cited in this review include experimental infections in animal models (mice, ferrets, guinea pigs, and chickens). Among these, ferret models are widely considered the gold standard for assessing mammalian transmissibility and pandemic potential, as they most closely recapitulate human influenza infection. In contrast, mouse and guinea pig models primarily inform relative virulence comparisons and viral replication kinetics rather than direct human infection risk. The conclusions of this review therefore draw on the convergence of evidence from multiple model systems, with greater weight given to ferret transmission studies and studies that include mechanistic validation, rather than relying on any single animal model.

Several previous reviews have provided valuable foundational overviews of H3 subtype AIV epidemiology and evolution [2,3,11]. Building on these contributions, the present review extends the current knowledge in four respects: (i) it integrates long-term surveillance data from wild birds, poultry, and live poultry markets across North America, Europe, and Asia, highlighting the global epidemiological patterns of H3 subtype AIV and the central role of live poultry markets in viral transmission and reassortment; (ii) it systematically analyzes the genomic composition and evolutionary pathways of H3N8 and H3N3 triple-reassortant viruses, and summarizes the reassortment features of other H3NX subtypes (including H3N6, H3N2, and H3N5), revealing the evolutionary patterns by which H3 subtype AIV continuously generates diverse genotypes through reassortment; (iii) it summarizes cross-species transmission events of H3 subtype AIV in multiple mammalian hosts (including dogs, cats, horses, pigs, seals, and humans), and synthesizes the coordinated effects of adaptive mutations across different gene segments on receptor-binding preference and pathogenicity; and (iv) it reviews current detection methods and vaccine platforms, discusses the challenges in current surveillance systems and diagnostic capacity, and proposes an integrated control framework to address these challenges.

Substantial systematic research has been conducted on the epidemiological characteristics, genetic evolution patterns, cross-species transmission mechanisms, and prevention and control technologies of H3 subtype AIV, with continuous improvements in scientific understanding. At the epidemiological level, H3 subtype AIV has a broad host spectrum, infecting at least 90 species of wild birds and poultry. Central Asia, Alaska, and the overlap zones between migratory bird flyways and poultry production in China represent key hubs for cross-border viral spread and persistent circulation. At the genetic evolution level, after H3 subtype AIV enters poultry populations, the hemagglutinin gene evolutionary rate increases significantly, and adaptive mutations continuously accumulate, accelerating the emergence of novel variants and reassortant strains. At the cross-species mechanism level, key receptor-binding site mutations such as HA-Q226L and HA-G228S can drive the switch in viral receptor preference from avian-type α-2,3 to human-type α-2,6 sialic acid receptors. This receptor preference remodeling is the core molecular basis mediating cross-host infection of AIVs in mammals and humans. Concurrently, novel reassortant strains, including H3N3 and H3N8, continue to emerge. Reassortant strains carrying specific H9N2-derived internal-gene constellations, particularly the full complement of internal-gene segments from G57-genotype H9N2 viruses, have been associated with enhanced mammalian adaptation in experimental models, although not all H9N2 gene combinations confer this advantage [99]. Notably, G25-genotype H3N8 viruses behind recent human spillover events have acquired this H9N2-derived internal-gene backbone of G57 origin [74].

In this review, the term “sustained transmission lineage” refers to viral populations capable of self-perpetuating circulation within a given host species, maintained by ongoing chains of host-to-host transmission without repeated reintroduction from external reservoirs. This distinguishes such enzootic circulation from isolated spillover events that do not generate onward transmission. For H3 subtype AIV, sustained transmission lineages have become established in horses (H3N8, dating to at least 1963) and dogs (H3N2, emerging around 2005), where viruses circulate endemically and continue to accumulate sequence variation via antigenic drift within these mammalian host populations [56,63]. This pattern differs from virus circulation in wild birds: although H3 viruses circulate broadly in migratory waterfowl, their dynamics are heavily shaped by seasonal migration, host aggregation, and frequent reassortment. They do not form host-restricted, independently sustained lineages analogous to those seen in horses and dogs; instead, they constitute a large, dynamically reshuffled gene pool distributed across diverse avian hosts [10,36]. This distinction is important for interpreting the differing public health implications of H3 circulation in wild birds versus mammalian hosts.

Functional studies have confirmed that point mutations such as PB2-D701N, M1-M192V, and PA-T97I can individually or synergistically reshape the replication efficiency, tissue tropism, and mammalian virulence of H3 subtype AIV. Among these, PB2-D701N is a core molecular marker enhancing cross-host pathogenicity [17,76]. However, current research has mainly focused on the effects of viral protein point mutations on in vitro replication efficiency and in vivo pathogenicity, with limited systematic analysis of the molecular mechanisms by which mutations mediate viral evasion of host innate immune responses. A novel influenza virus immune evasion pathway mediated by the FGF8–TRIM16–RIG-I axis has recently been identified [100]. Mechanistically, FGF8 serves as a molecular scaffold, recruiting the E3 ubiquitin ligase TRIM16 and RIG-I to form a complex that mediates K48-linked ubiquitination and proteasomal degradation of RIG-I, thereby blocking activation of the downstream type I IFN signaling pathway. Cellular experiments confirmed that TRIM16 silencing significantly inhibited influenza A replication and restored IFN-β secretion levels. This study was the first to reveal that influenza A viruses can hijack the FGF8–TRIM16 axis to achieve host innate immune evasion, providing a novel molecular perspective for understanding virus–host interactions [100]. Notably, the conservation of this pathway has only been validated in H9, H13, and other AIV subtypes; functional validation has not yet been performed for circulating H3 subtype AIVs, representing an important direction for subsequent systematic analysis of cross-host immune evasion mechanisms.

Regarding vaccine development, multiple novel vaccine platforms have been applied to H3 subtype AIV research, providing solid technical support for epidemic prevention and emergency stockpiling. The CK/NT308/H3N3 candidate strain identified through traditional inactivated vaccine platform screening provides complete clinical protection against currently predominant H3N3 and H3N8 strains [23]. Concurrently, novel technology platforms—including adenovirus-vectored and mRNA vaccines—offer advantages such as short development cycles, strong immunogenicity, and comprehensive protection. They compensate for the limitations of traditional vaccines, which suffer from slow iteration and limited strain matching. This provides novel technological pathways for emergency prevention and proactive vaccine stockpiling against H3 avian influenza [81,96].

Although basic research and control technologies for H3 subtype AIV have achieved breakthroughs, significant gaps remain in the comprehensive prevention and control system, and the overall situation remains severe. Currently, global surveillance for influenza A viruses through existing frameworks can detect H3 subtype AIV. However, given the increasing cross-species transmission potential of H3 viruses, continued efforts to strengthen surveillance for influenza A viruses with zoonotic risk—particularly those carrying mammalian-adaptive molecular markers—are warranted. This makes it difficult to promptly capture viral variant dynamics, covert transmission characteristics, and cross-species transmission risks. Consequently, preemptive early warning and precision control capabilities remain insufficient. At the same time, H3 subtype AIV can persist and continuously evolve in LPM environments. It has also established stable adapted lineages in multiple mammalian species, including dogs and horses, creating persistent animal reservoirs. This substantially increases the difficulty of preventing repeated viral spillover, continuous variation, and widespread transmission.

Drawing upon the molecular evidence compiled throughout this review, a conceptual tiered early-warning framework is outlined as a working model requiring further empirical evaluation and refinement. Tier 1 (Surveillance Alert) may suggest elevated risk for occasional spillover events when HA receptor-binding mutations such as Q226L or G228S are detected, given their known capacity to alter viral host tropism. Tier 2 (Public-health consideration) may be invoked when these HA mutations co-occur with polymerase-associated mammalian-adaptive markers such as PB2-E627K or PB2-D701N; such combined molecular signatures have been associated in experimental studies with enhanced mammalian replication capacity and potential for more efficient cross-species transmission. This conceptual framework can serve as a preliminary reference for prioritizing H3-positive isolates for deeper virological characterization. Nevertheless, its predictive performance and practical threshold criteria remain incompletely defined and will require systematic validation using both experimental datasets and real-world surveillance observations.

Looking forward, three priority directions are identified for mitigating the public health risk posed by H3 subtype AIV. First, strengthening global surveillance networks by integrating wild bird, poultry, and mammalian monitoring under a One Health framework is urgently needed to capture viral dynamics and early spillover signals. Second, addressing diagnostic gaps at primary laboratories through capacity building and deployment of field-deployable rapid detection tools will improve early warning capabilities. Third, systematic evaluation of zoonotic potential using experimental models—including receptor-binding assays, animal pathogenicity studies, and airborne transmission models—is essential to assess the pandemic risk of emerging H3 reassortants and to guide preparedness efforts.

## Figures and Tables

**Table 1:** Distribution of H3 subtype AIV in different hosts.

Subtype	Host(s)	Reference(s)
H3N1	Wild birds	[11]
	Chickens	[15,16]
H3N2	Wild birds	[17]
	Chickens, ducks, etc.	[2,18]
	Dogs, cats, pigs	[12,19]
H3N3	Wild birds	[11]
	Chickens, ducks, pigeons	[20,21]
	Mice, guinea pigs (experimental infection)	[20,21]
H3N4	Wild birds	[22]
H3N5	Wild birds	[11]
	Chickens, geese	[23]
H3N6	Wild birds	[22]
	Ducks, geese	[24,25]
H3N7	Wild birds	[11]
H3N8	Wild birds	[11,22]
	Chickens	[26,27]
	Dogs	[14]
	Pigs (experimental infection)	[28]
	Seals	[7,29]
	Horses	[22]
	Humans	[7]

**Table 2:** Key mammalian adaptive mutations in H3 subtype AIV and its derivative viruses (H3N2 CIV).

Key Amino Acid Substitution	Phenotypic Change/Function	Reference(s)
Polymerase and internal genes		
PB2-E627K	Emerged as an adaptive mutation during mammalian passage of H3N8 subtype AIV in ferrets; enhances polymerase activity and transmissibility	[27]
PB2-D701N	Promotes replication of waterfowl-origin H3N2 subtype AIV in mammalian models; enhances viral pathogenicity	[17]
PB2-K318R	Upregulates polymerase activity of H3N8 subtype AIV; enhances viral virulence and mammalian host adaptation	[49,50]
PB2-I292T	Key mutation for efficient H3N2 CIV colonization in dogs	[70]
PB2-G590S	Key mutation for efficient H3N2 CIV colonization in dogs	[70]
PB2-S107N	Highly homologous to human seasonal H3N2 influenza virus; narrows the molecular adaptation gap between avian-origin and human influenza viruses	[67]
PB1-F2-N66S	Upregulates polymerase activity of H3N8 subtype AIV; enhances viral virulence and mammalian host adaptation	[49,50]
M1-A227T	Highly homologous to human seasonal H3N2 influenza virus; narrows the molecular adaptation gap between avian-origin and human influenza viruses	[67]
M1-M192V	Promotes replication of waterfowl-origin H3N2 subtype AIV in mammalian models; enhances viral pathogenicity	[17]
HA receptor-binding sites		
HA-Q226L	Reshapes receptor-binding pocket; drives H3N8 receptor preference switch from avian-type SAα2,3-Gal to human-type SAα2,6-Gal	[71]
HA-G228S	Reshapes receptor-binding pocket; drives H3N8 receptor preference switch from avian-type SAα2,3-Gal to human-type SAα2,6-Gal	[71]
HA antigenic sites		
HA-D97N	H3N2 CIV antigenic drift-associated sites; continuously undergo amino acid substitutions during long-term dog circulation, facilitating escape from host immune pressure	[70]
HA-A176T	H3N2 CIV antigenic drift-associated sites; continuously undergo amino acid substitutions during long-term dog circulation, facilitating escape from host immune clearance	[70]
HA-N204D	H3N2 CIV antigenic drift-associated sites; continuously undergo amino acid substitutions during long-term dog circulation, facilitating escape from host immune clearance	[70]
HA-V212I	H3N2 CIV antigenic drift-associated sites; continuously undergo amino acid substitutions during long-term dog circulation, facilitating escape from host immune clearance	[70]
HA-W237L	H3N2 CIV antigenic drift-associated sites; continuously undergo amino acid substitutions during long-term dog circulation, facilitating escape from host immune clearance	[70]

**Table 3:** Comparison of diagnostic methods for H3 subtype AIV detection.

Method	Target	Detection_Limit	Sensitivity	Specificity	Time	Sample_Type	Field_Applicability	Note
Multiplex RT-PCR (GeXP)	HA genes	100 copies	High	High	2–4 h	Swabs/tissues	Laboratory	Ref. [83]
Triplex Real-time PCR	H3, H4, H5 subtypes	2.1 × 10^2^ copies/uL	High	High	2–3 h	Swabs/tissues	Laboratory	Ref. [84]
Quantum Dot Strip	HA1 protein	15.63 ng/mL	Moderate	High	15 min	Swabs	Field/On-site	Ref. [85]
Double-Ab Sandwich ELISA	H3 antigen	Not specified	Moderate	High	2–3 h	Swabs/sera	Laboratory	Ref. [86] (LOD not provided)
RT-RAA	HA gene	224 copies/reaction	High	High	30 min	Swabs	Primary labs	Ref. [87]

## Data Availability

No new data were created or analyzed in this study. Data sharing is not applicable to this article.

## Abbreviations

The following abbreviations are used in this manuscript:

AIV	Avian influenza virus
CIV	Canine influenza virus
CRISPR	Clustered regularly interspaced short palindromic repeats
ELISA	Enzyme-linked immunosorbent assay
FGF8	Fibroblast growth factor 8
HA	Hemagglutinin
HPAIV	Highly pathogenic avian influenza virus
IFN	Interferon
ISG	Interferon-stimulated gene
LNP	Lipid nanoparticle
LPAIV	Low-pathogenicity avian influenza virus
LPM	Live poultry market
MDCK	Madin–Darby canine kidney
MLD_50_	Mouse median lethal dose
mRNA	Messenger RNA
NA	Neuraminidase
RNP	Ribonucleoprotein
RT-PCR	Reverse transcription polymerase chain reaction
RT-RAA	Reverse transcription recombinase-aided amplification
SPF	Specific pathogen-free
WOAH	World Organisation for Animal Health
WHO	World Health Organization
